# Implicit measurement of emotional experience and its dynamics

**DOI:** 10.1371/journal.pone.0211496

**Published:** 2019-02-05

**Authors:** Esther Eijlers, Ale Smidts, Maarten A. S. Boksem

**Affiliations:** Department of Marketing Management, Rotterdam School of Management, Erasmus University, Rotterdam, The Netherlands; Victoria University of Wellington, NEW ZEALAND

## Abstract

Although many studies revealed that emotions and their dynamics have a profound impact on cognition and behavior, it has proven difficult to unobtrusively measure emotions. In the current study, our objective was to distinguish different experiences elicited by audiovisual stimuli designed to evoke particularly happy, sad, fear and disgust emotions, using electroencephalography (EEG) and a multivariate approach. We show that we were able to classify these emotional experiences well above chance level. Importantly, we retained all the information (frequency and topography) present in the data. This allowed us to interpret the differences between emotional experiences in terms of component psychological processes such as attention and arousal that are known to be associated with the observed activation patterns. In addition, we illustrate how this method of classifying emotional experiences can be applied on a moment-by-moment basis in order to track dynamic changes in the emotional response over time. The application of our approach may be of value in many contexts in which the experience of a given stimulus or situation changes over time, ranging from clinical to consumption settings.

## Introduction

Emotions are fundamental in guiding our behavior; they are indices of events that we value or desire to different extents in our everyday lives [1]. Numerous studies have shown that emotions have a profound impact on cognition: emotions modulate attention [2] [3] and enhance memory for valuable events [4] [1] in order to better predict occurrences of such events in the future. Emotions also influence social and economic decision-making [5] [6] [7] by acting as a motivator [8] [7], by providing information [9] [7], and by influencing the way we interact with others [10].

However, the actual measurement of emotions has proved to be challenging. Emotion ratings acquired through self-report can potentially be distorted because of social desirability concerns [11]. That is, people may *not want to* express exactly how they feel. Even in the absence of these factors, it has been shown that people are very limited in their ability to reflect on their internal mental processes and to accurately report on these processes [12]. That is, they may *not even be able to* put their feelings into words accurately. Indeed, as affective processes largely occur outside our awareness [13], emotions do not have to be experienced consciously to influence judgement and behavior [14]. In addition, the task of consciously reporting on one’s (unconscious) emotional state may actually change this state, potentially changing the relationship between emotion and subsequent behavior [15] [16].

Neuroimaging methods may provide a solution to this problem by recording brain activity underlying both conscious and unconscious processes, without the need to consciously and cognitively reflect on them [17]. Functional magnetic resonance imaging (fMRI) has been employed successfully to localize neural networks involved in many cognitive processes such as working memory or valuation of choice alternatives, while participants perform a task that engages one of these specific processes implicitly (see [18] and [19] respectively for meta-analyses). There also have been several fMRI studies in which the neural correlates of emotions are explored (see [20] for a meta-analytic review). However, multiple meta-analyses have shown that there is little evidence for activity in any single brain region to be consistently and specifically associated with a specific emotion. This is why a multivariate pattern analysis (MVPA) approach has been suggested to be more appropriate for investigating emotions; to allow for the search of neural activation patterns that occur distributed (but simultaneously) across the brain ([21], but see [22]).

While the core advantage of fMRI is providing insight into the particular brain structures involved, it is less useful for gaining insight into how these neural processes evolve over time. Emotions are transient experiences [23], and people’s (intensity of their) experienced emotions are subject to change under the influence of the external environment [24]. The dynamics of the emotional experiences have a critical impact on the subsequent (behavioral) response: People do not assess an affective experience based on the average experience, but instead rely heavily on the intensity of peak and final moments, also referred to as the peak-end rule [25] [26]. It would therefore be highly valuable to be able to decode and monitor discrete emotional responses relatively unobtrusively on a moment-by-moment basis. Being able to accurately measure the dynamics of emotions would serve many practical purposes in contexts such as media consumption, gaming, online buying, and other aspects of consumer experience, but also in clinical settings in which one is concerned with changes of the patient’s emotions over time.

Electroencephalography (EEG) is a suitable alternative to fMRI against this background, with a lower spatial resolution but with a much higher temporal resolution. With EEG, the fluctuations in voltage that are measured by electrodes at the scalp reflect the summed activity of large, synchronously active, populations of neurons at the surface of the brain [27]. This (in combination with volume conduction) precludes accurate localization of the source of the measured activity. However, because electrical activity is measured directly (as opposed to via the hemodynamic response as with fMRI), the temporal resolution is retained.

Studies in the past decades have shown that oscillations in different frequency ranges or so-called frequency bands of the EEG signal, relate to specific psychological processes in the brain [28] (see [29] for review). With regard to emotions, early EEG studies (e.g., [30]) have investigated positive versus negative affective experiences using the asymmetry in oscillatory activity between hemispheres. Although the initial studies suggested that greater left than right frontal activity was associated with the experience of positive affect, and greater right than left frontal activity with the experience of negative affect [31], later studies revealed that the underlying factor was motivational direction (i.e., approach and withdrawal rather than positive and negative affect, respectively) (see [32] for review).

Going beyond emotional valence, measuring more specific emotions would provide more detailed information regarding an elicited response and its potential behavioral consequences. However, clear EEG correlates of specific emotional experiences have so far not been conclusively shown. As with fMRI, it is unlikely that specific emotions are associated with each their own particular EEG component. The aim of our study is therefore to use a multivariate approach in order to search for patterns of frequency distributions in the EEG data that distinguish different emotional experiences. In the current study, these experiences were elicited by audiovisual stimuli designed to evoke particularly happy, sad, fear and disgust emotions. It should be noted that we not necessarily measure the specific emotions happy, sad, fear, and disgust (if they exist), but rather *representations of emotional experiences*, as elicited by audio-visual stimuli, that can be grouped together and labeled as such. Thus, we use happy, sad, fear and disgust merely as descriptive labels for particular experiences as elicited by audio-visual stimuli.

In our multivariate approach we based supervised classification of the emotion categories on activity that distinguishes between emotions categories. Importantly, we do not make a priori assumptions about which features of the signal (frequency bands or scalp topography) would be predictive of distinctions between emotional experiences. The advantage of this method is that we will be able to interpret the observed differences between emotional experiences, based on the patterns of frequencies and their topography, in terms of underlying processes that are known to be associated with these activation patterns.

We elicited the specific emotional experiences by displaying short videos that we selected for this purpose, as dynamic multimodal audiovisual stimulation represents the best and most natural way of eliciting emotions [24][33]. Participants viewed five short clips for each of the four emotions under investigation, while their EEG was recorded. We then classified the emotional content of these clips based on the features (frequency and topography) of the EEG signal. Finally, we illustrate that the method we applied to classify emotional experiences can be used to track dynamic changes in the emotional response over time.

## Methods

### Participants

We recruited 40 students from the university population. They all had normal or corrected-to-normal vision and had no history of neurological illness. Before the experiment, written informed consent was obtained, and participants received 25 Euros for their participation. Three participants were excluded from the analysis because of excessive artefacts in the reference channels and/ or channels recording the eye movements, precluding appropriate pre-processing of the data. The final sample therefore consisted of 37 participants (24 female) between 18 and 28 years (*M* = 22.2, *SD* = 2.6) of age.

### Stimuli

We selected videos that would elicit a strong emotional response in the participants according to an expert panel. The content of the videos consisted of scenes from movies or documentaries and were selected to elicit one specific emotional experience (see Appendices A and B in S1 Supplementary Material for details). The length of the video clips ranged from 22 seconds to 200 seconds (*M =* 96.2 s, *SD* = 36.9 s). More specifically, the happy videos had a mean duration of 112.2s (SD = 54.6), the sad videos 118.0s (SD = 14.3), the fear videos 82.0s (SD = 32.6), the disgust videos 58.0s (SD = 23.2). The videos eliciting happy and sad responses were relatively longer in duration than the videos eliciting fear and disgust, because eliciting happiness or sadness requires in general more time to build up in a context, whereas disgust and fear responses are more immediate without much need for context (see Appendix D in S1 Supplementary Material for robustness check 1 in which the analyzed segments have equal durations across emotion conditions).

We included a video clip from the beginning of the animated movie *Up* specifically to illustrate tracking of the emotional response over time, because this clip comprises a complete storyline (i.e., a summary of the lives of a man and woman that get together). In the first and main part of this video the content is predominantly happy, but at a certain point in the video the happy content clearly ceases to dominate while the sad content increases, allowing us to demonstrate content validity of our method when we track the emotional response over time.

### Procedure

The Erasmus Research Institute of Management (ERIM) Internal Review Board granted approval to conduct the experiment (2016/04/26-44486mvb). The participants received written and verbal instructions on the task that they were going to perform upon arrival at the lab. Participants were unaware of the purpose of the study, but they were made aware that the videos that they were going to watch included content from the genres action, comedy, crime, horror, thriller, romance, drama, mystery and musical. We asked the participants to empathize with the people in the videos as much as possible, stay attentive and enjoy watching the videos. We notified participants beforehand of the presence of some intense scenes from movies and TV series. We did not mention that we would ask them to complete a questionnaire about the videos after the EEG recording.

During the EEG data collection, participants were seated in a slightly reclining chair positioned in front of a 19-inch PC monitor in a sound-attenuated, electrically shielded, dimly lit room. After showing the instructions again on the screen, the videos were presented in blocks, with each block consisting of five videos belonging to one of the four emotion categories happy, sad, fear or disgust. We reasoned that a block design was the best approach in order to induce and maintain the emotional experience optimally, rather than a design with rapid and constant switching between emotions. We randomized the order of the blocks as well as the videos within blocks, across participants. Between each block, a neutral video that contained part of a documentary was presented in order to return to a neutral or baseline emotional state. The videos were presented at a resolution of 1280 x 720, and the inter-stimulus interval, consisting of a black screen, was three seconds.

To verify the videos’ effectiveness in eliciting the specific emotional responses in our participants, we asked participants to complete a questionnaire about the previously viewed videos after we finished the EEG data collection. Participants had to indicate for each video the extent to which they, respectively, felt happy, sad, fear, and disgust during the video on a scale from one (felt not at all e.g., happy) to five (felt extremely e.g., happy). In order to aid the recollection of (the experience of) the video, we provided a screenshot of a characteristic scene from that video before the question. The video screenshots and questions about the videos were presented in random order (i.e., not in blocks per emotion).

### EEG recording and analysis

The EEG data was acquired using the BioSemi Active Two system with 64 active Ag-AgCl electrodes. Additional flat type electrodes were placed on the right and left mastoid, and in the eye region in order to record eye movements or electro-oculograms (EOGs): Electrodes were placed below and above the left eye in line with the pupil to record vertical EOGs, and at the outer canthi of both eyes to record horizontal EOGs. The EEG and EOG signals were sampled at a rate of 512 Hz. All preprocessing was done in Brain Vision Analyzer software (BVA; Brain Products). The data was first down-sampled to 256 Hz, then re-referenced to the averaged mastoids, and filtered with a low cutoff filter of 1 Hz with a slope of 48 dB/octave and a notch filter of 50 Hz. Thereafter, the data was segmented into 25 segments (one for each video), with segments lasting from the beginning to the end of the video. We then split the segments further into 50% overlapping segments of 256 data points. We applied Gratton and Coles ocular correction as implemented in BVA, and standard artifact detection and rejection criteria where segments were rejected that contained jumps larger than 30μV/ms, amplitude differences exceeding 150μV/ 200ms, and amplitude differences below 0.5 μV/ 100 ms. Note that only the channels that contained artifacts were deleted within the given segment, and not the entire segment. Then, data was decomposed into different frequencies (1–128 Hz) using a Fast Fourier Transform (FFT, using a 100% hanning window). Finally, we averaged the frequency data across all segments for each video, and for each participant separately. The resulting frequency data was exported to Matlab (Mathworks). Note that this results for each video in averaged frequency data across the entire video duration (but for each electrode, for each frequency), since our DV (emotion category labels) is also at the video level.

For the initial phase of the analysis described below, we only used the first part of the *Up* video for the representation of a happy emotional response. Based on the predominantly happy content of the first part of the video, we averaged frequency data across the first 200 seconds of the video. In order to track the emotional response over time during the complete video, we additionally exported the non-averaged frequency-domain data for the entire *Up* video per second (261 seconds in total).

### Statistical analyses

After transforming the EEG data obtained during viewing of the videos to the frequency domain, we standardized (i.e., z-transformed) the data for every participant, electrode, and frequency across all videos. Further analyses consisted of two parts (one for classifying the emotional experiences happy, sad, fear, and disgust that were elicited by viewing videos, based on the patterns of frequency distributions observed in the EEG data, and one for illustration of tracking of the emotional response over time), each with multiple stages (see Appendix C in S1 Supplementary Material for more details on the statistical analyses).

For the first part, classifying the emotional experiences, we started with feature selection: Using a subset of the observations (i.e., a subset of the videos), we selected features (i.e., electrode-frequency combinations) that were most informative in distinguishing the specific emotions in order to reduce the dimensionality of the data. Per participant, electrode, and frequency, each emotion was contrasted with the average of the other three emotions. For each emotion then, one-sample *t*-tests across participants were applied to determine the 10% most informative features to use for classification (see Appendix E in S1 Supplementary Material for similar results with 5% and 20% features: robustness check 2). Thereafter, we proceeded with training and testing the classifiers. That is, with the remaining observations (i.e., those not used for feature selection), we trained support-vector machines (SVMs; six two-class classification models for the six combinations of four emotions, and also a multi-class model) on the selected features to generalize the distinction between emotional responses to new data, using cross-validation. We repeated feature selection and classifier training and testing 500 times, with for each repetition a different random subset of observations used for feature selection and thus also for the training and testing stage in order to rule out a selection bias as explanation of our results (see Appendix G in S1 Supplementary Material for robustness checks regarding the number of repetitions).

For the second part of the analysis, focused on tracking the emotional response over time, we applied a newly trained classifier to the complete video from the animated movie *Up*. We first performed feature selection and training of a classifier on happy, sad, fear and disgust emotional experiences elicited by the videos (i.e., computed a multi-class model), but this time we excluded the happy video *Up*, as well as for each other emotion the video with the lowest average rating on the emotional response it should have elicited. The rest of the analysis was similar to the analysis in part one, except for the final testing stage that was now replaced by a prediction stage. In the prediction stage, we used the trained classifier to compute the posterior probabilities that the emotional response was happy, sad, fear or disgust for every second of the *Up* video, averaged across participants. Since the content of the video becomes less happy over time (after approximately 200 seconds), and the reverse holds for the sad content, we show the contrast between the probability that the response is classified as happy versus sad.

## Results

Participants indicated for each video the extent to which they felt happy, sad, fear, and disgust during viewing the video, after we finished the EEG data collection. This enabled us to verify the videos’ effectiveness in eliciting the specific emotional responses in our participants (i.e., manipulation check). Based on the results of the manipulation check (see Appendix C for details on statistical analyses and Appendix H for results in S1 Supplementary Material), we concluded that the emotional responses that the videos targeted to elicit, are indeed the emotions that the participants predominantly experienced during viewing of the videos. These results suggest that the EEG activity averaged across the duration of the videos, is representative of a happy, sad, fear, and disgust response, respectively, and that we can use this data to functionally localize specific emotion-related activity patterns.

### Classifying the emotional experiences based on EEG data

#### Feature selection

**Fig 1:**
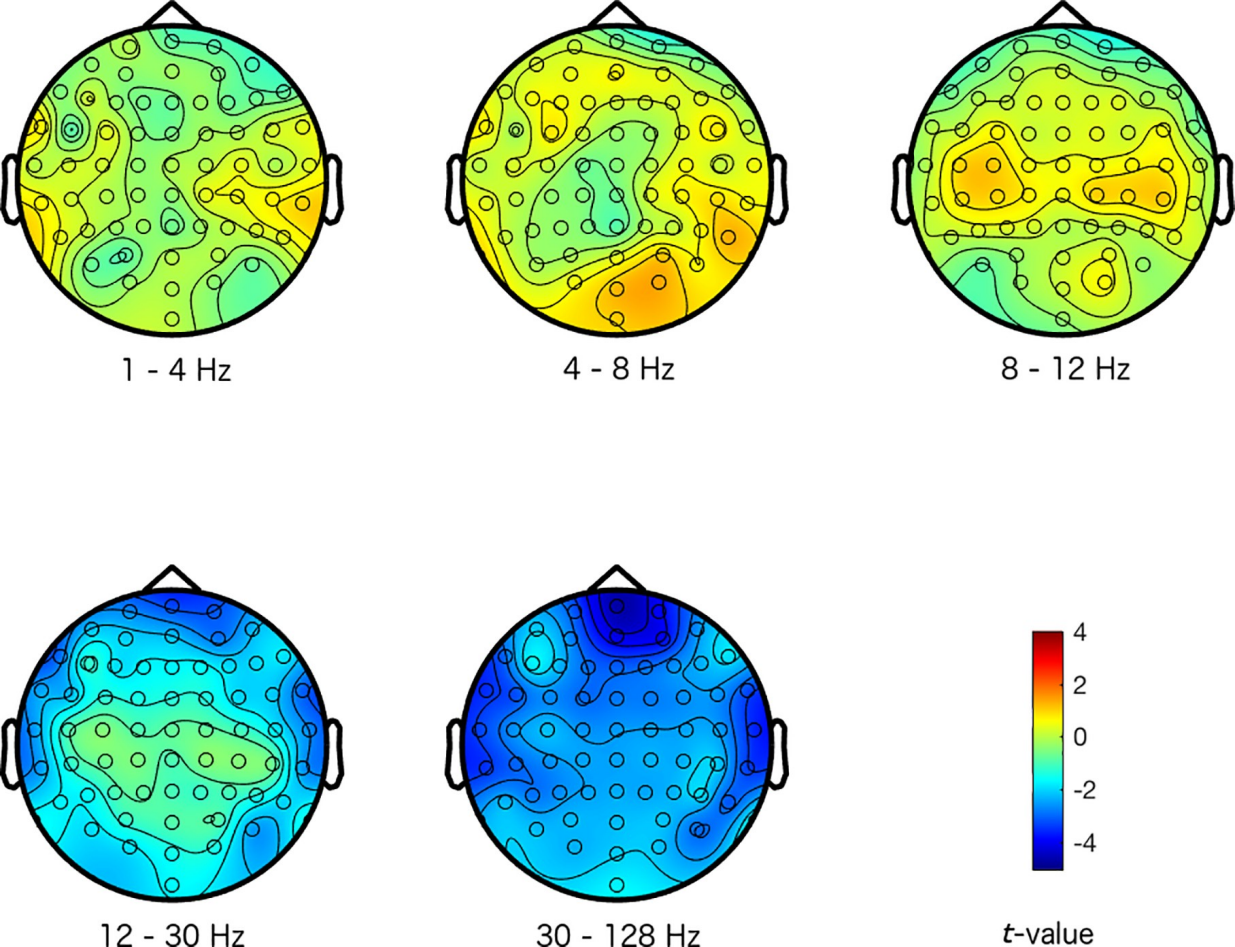
Maps of the difference between a happy response and the other emotional responses. The colors represent *t*-values. The different scalp maps show the contrast (expressed in *t*-values) between activity representing a happy response, and activity representing the other emotional responses for the specific frequencies that are indicated below the maps (the delta (1–4 Hz), theta (4–8 Hz), alpha (8–12 Hz), beta (12–30 Hz), and gamma (30–128 Hz) frequency range respectively), and across the head for the 64 electrodes.

**Fig 2:**
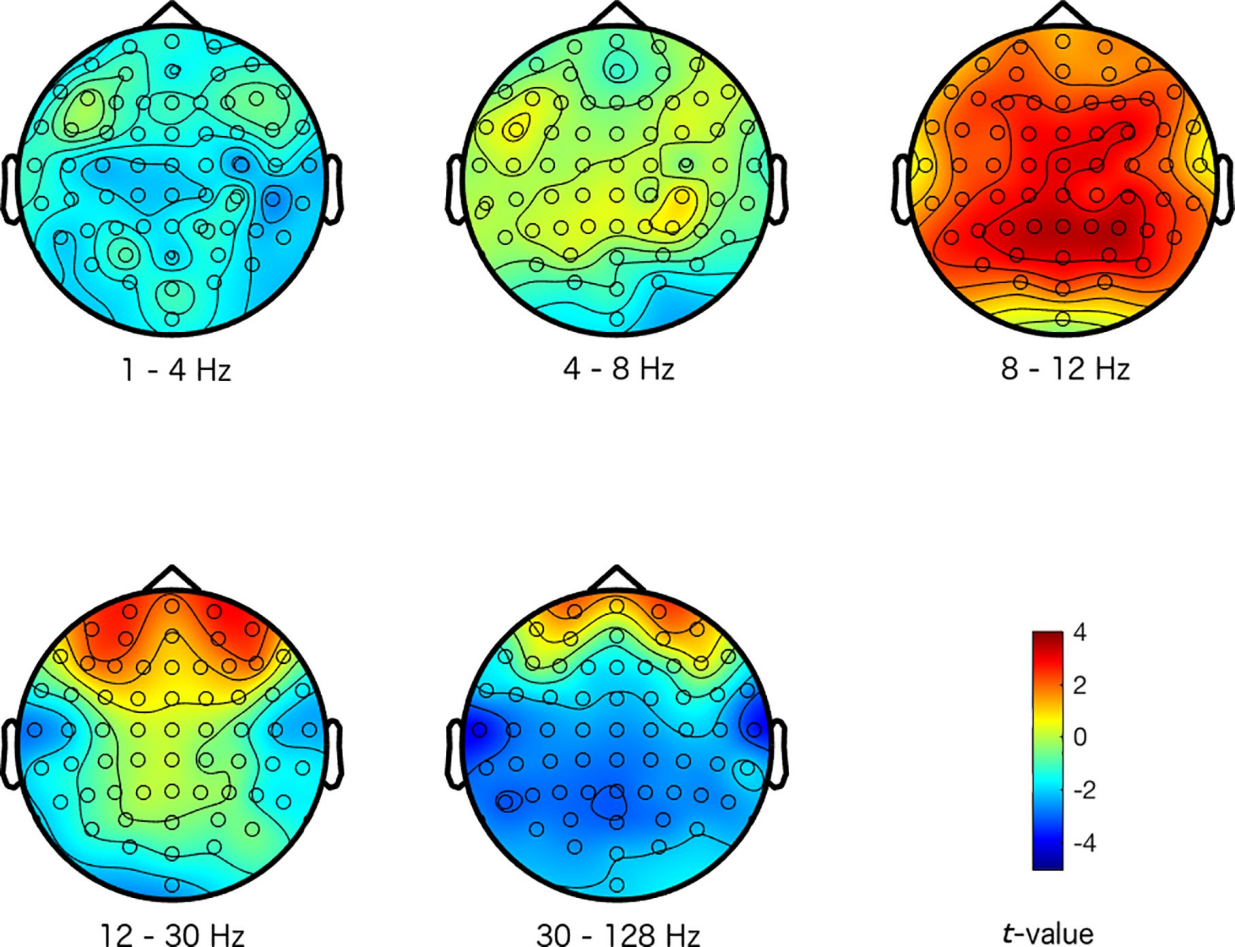
Maps of the difference between a sad response and the other emotional responses. The colors represent *t*-values. The different scalp maps show the contrast (expressed in *t*-values) between activity representing a sad response, and activity representing the other emotional responses for the specific frequencies that are indicated below the maps (the delta (1–4 Hz), theta (4–8 Hz), alpha (8–12 Hz), beta (12–30 Hz), and gamma (30–128 Hz) frequency range respectively), and across the head for the 64 electrodes.

**Fig 3:**
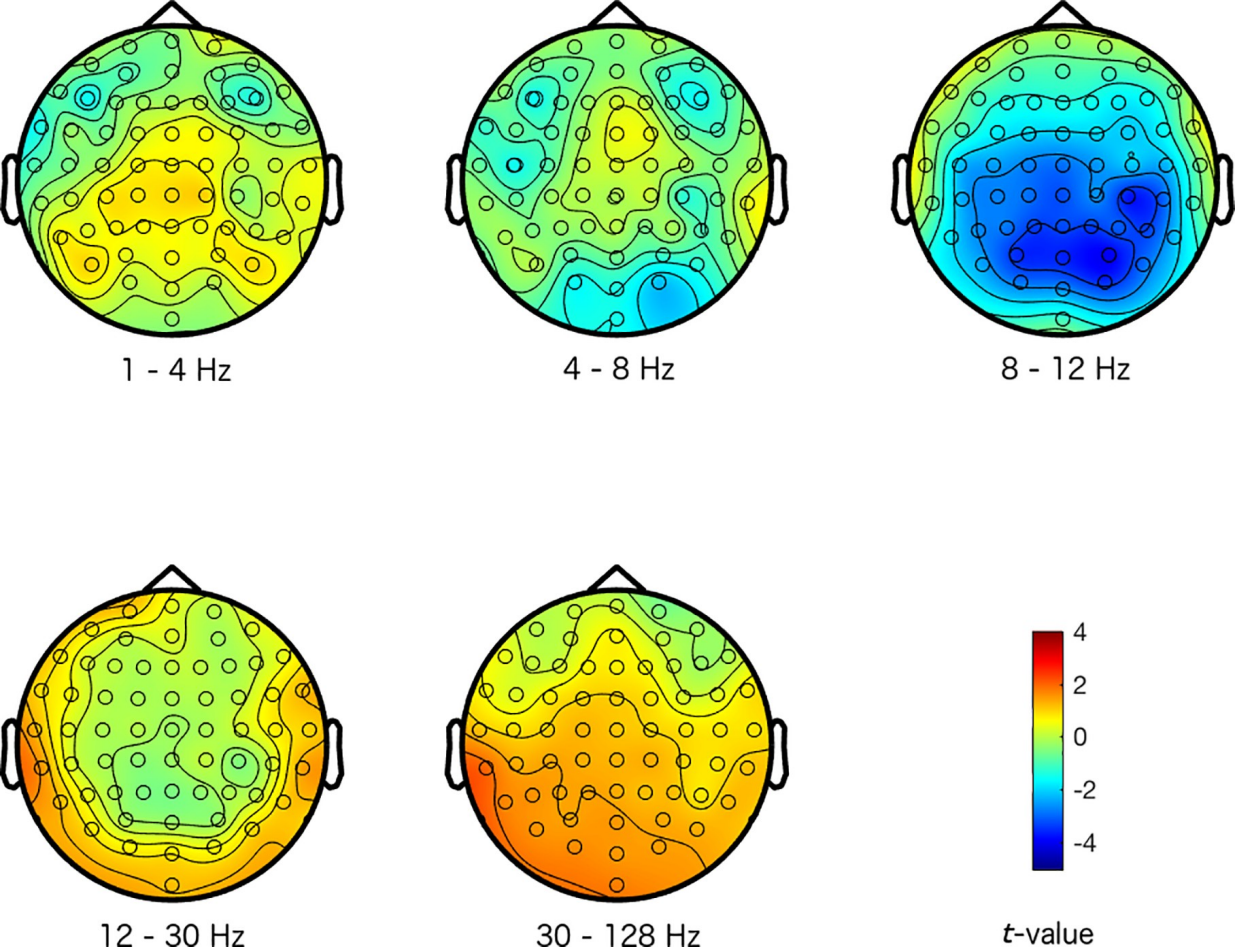
Maps of the difference between a fear response and the other emotional responses. The colors represent *t*-values. The different scalp maps show the contrast (expressed in *t*-values) between activity representing a fear response, and activity representing the other emotional responses for the specific frequencies that are indicated below the maps (the delta (1–4 Hz), theta (4–8 Hz), alpha (8–12 Hz), beta (12–30 Hz), and gamma (30–128 Hz) frequency range respectively), and across the head for the 64 electrodes.

**Fig 4:**
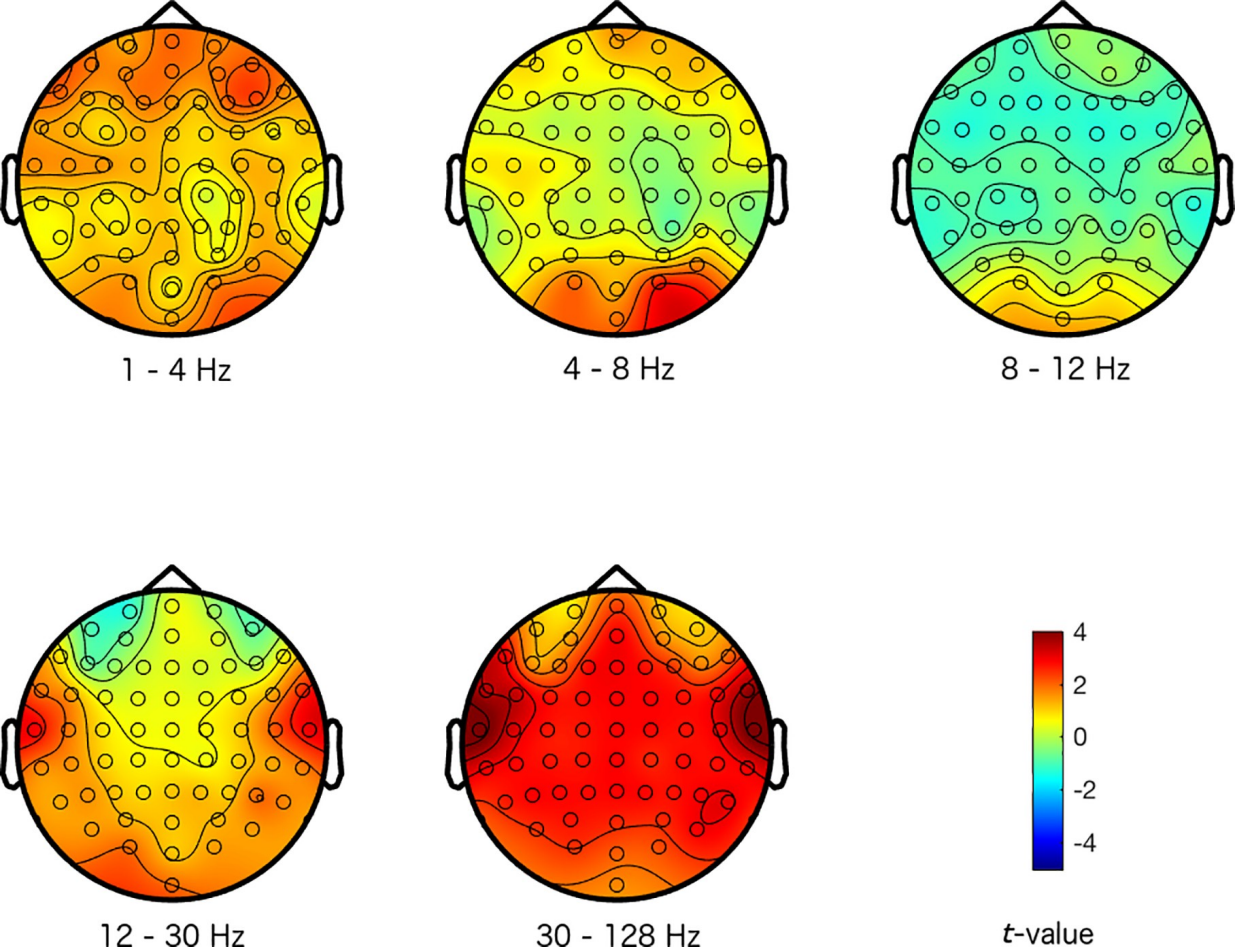
Maps of the difference between a disgust response and the other emotional responses. The colors represent *t*-values. The different scalp maps show the contrast (expressed in *t*-values) between activity representing a disgust response, and activity representing the other emotional responses for the specific frequencies that are indicated below the maps (the delta (1–4 Hz), theta (4–8 Hz), alpha (8–12 Hz), beta (12–30 Hz), and gamma (30–128 Hz) frequency range respectively), and across the head for the 64 electrodes.

The magnitude and sign of the *t*-values, averaged across the 500 repetitions, indicate how a specific emotional response differentiated from the other emotional responses at the different electrode-frequency combinations (see Figs 1–4). Note that we did not group the data into corresponding frequency bands in any of the analysis stages, but we merely did so here to provide an interpretable structure to the figures and results.

Inspecting Figs 1–4, suggests that the happy and disgust response differentiated most strongly from the other emotional responses in the higher frequency ranges. Happy was mostly associated with decreased gamma activity at frontal and temporal sites, and disgust was associated with increased gamma at temporal areas. The sad response most strongly differentiated from other emotional responses with more alpha activity present across the scalp. Finally, the fear response differentiated most clearly from other emotional responses in the alpha frequency range, with reduced alpha predominantly at centro-posterior sites (see Figs 1–4 for more detailed differences between the emotions).

Across the 500 repetitions of the complete analysis, slightly different features were selected, and thus also used in the stages of training and testing the classifiers (see Appendix I in S1 Supplementary Material for how often the specific features were selected across the repetitions).

#### Classifier training and testing

Computing the out-of-sample generalization accuracy for all 500 repetitions, resulted in a distribution of accuracies indicating generalizability of the distinction between emotions to new data, for the seven classifiers (see Table 1).

**Table 1:** Mean and percentiles for the distributions of out of sample generalization accuracies across 500 repetitions.

Classifier		Mean	Min.	2.50%	25%	50%	75%	97.50%	Max.
Fear	Disgust	71.37	60.36	64.86	69.37	71.62	73.42	77.48	79.73
Sad	Disgust	81.54	73.87	77.03	80.18	81.53	83.33	86.04	88.74
Sad	Fear	74.56	66.67	68.47	72.52	74.32	76.58	80.18	83.78
Happy	Disgust	77.05	68.92	71.62	75.23	77.03	79.28	81.98	85.59
Happy	Fear	76.14	67.57	70.72	74.32	76.13	77.93	81.53	85.14
Happy	Sad	78.18	70.27	72.52	76.13	77.93	80.18	83.33	86.49
Multi-class (all 4 emotions)	57.54	49.55	52.48	55.86	57.66	59.23	61.94	64.86

Since the models were trained on an equal number of category members (videos per emotion), theoretical chance level accuracy was 50% for the two-class models, and 25% for the multiclass models. The ability of the classifiers to generalize the distinction between emotions to new data was well above chance level, with the fear and disgust response being the most difficult to distinguish (median 71.62% out of sample generalization accuracy) and the sad and disgust response being the easiest to distinguish (median 81.53% out of sample generalization accuracy). The multi-class classifier was also well able to generalize the distinction between all four emotions to new data with a median accuracy of 57.66% compared to chance level of 25% (see Appendix J in S1 Supplementary Material for significant differences from benchmarks created by permuting emotion labels, which were approximately similar to theoretical chance levels). Although we did not intend to classify a ‘neutral response’ from the neutral videos that were presented between emotion blocks, including the neutral category in the classifiers yielded similar results (see Appendix F in S1 Supplementary Material for robustness check 3).

### Illustration of tracking the emotional response over time

**Fig 5:**
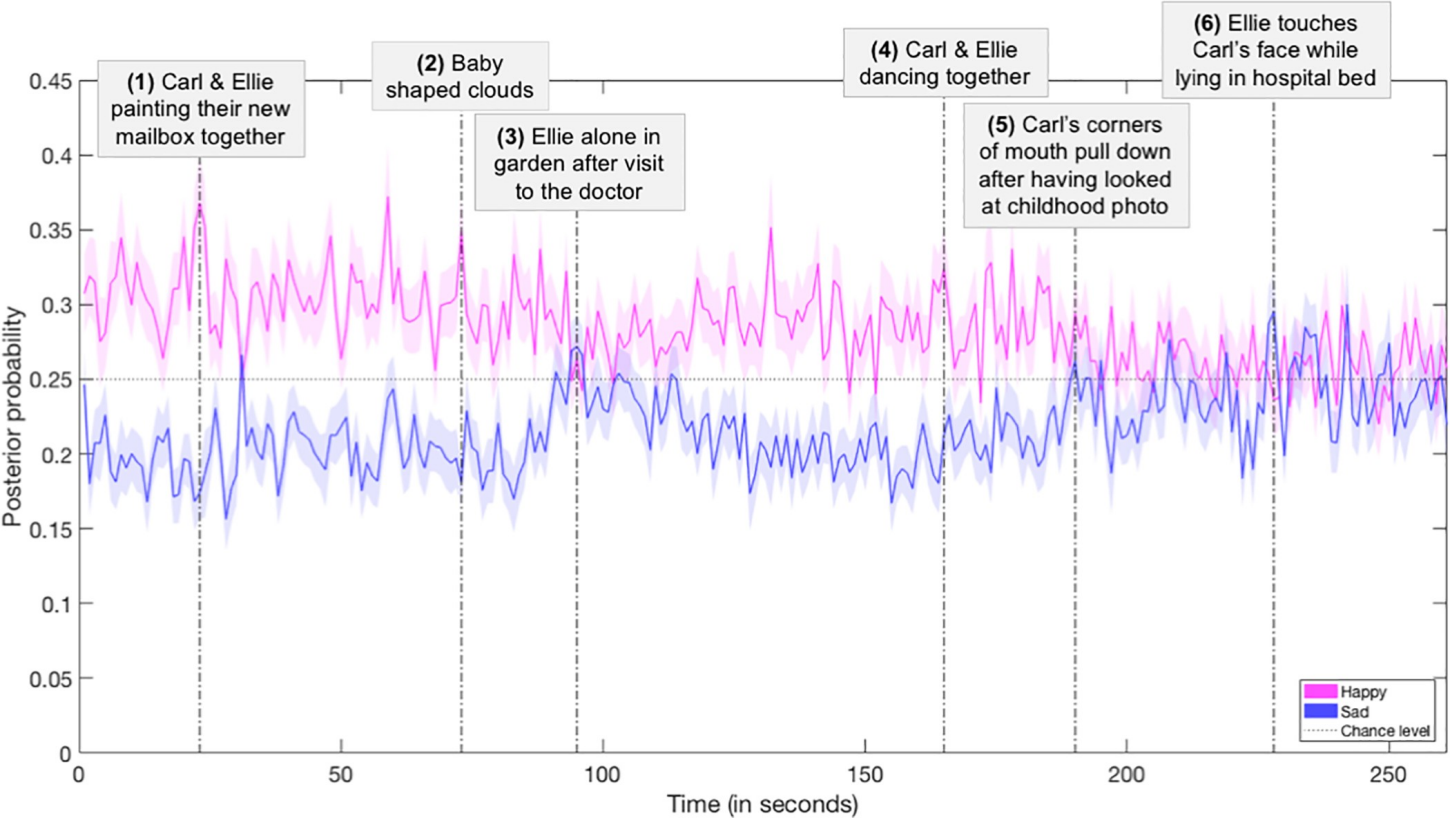
Dynamics of posterior probabilities for *Up*. Averaged across participants and 500 repetitions, with different observations used in the feature selection and classifier training stages across repetitions. The shaded areas indicate the standard deviation across repetitions. The vertical lines illustrate six examples of scenes at different moments in time, with moments (3), (5) and (6) indicating parts of the video that contain relatively more sad content. (1) Carl and Ellie just got married and are renovating the house: the posterior probabilities for a happy response are high. Once in a while they go on a picnic and look at the sky full of clouds. First, they see a cloud turn into an animal, then they see a cloud turn into a baby, and eventually at (2) all the clouds start to look like babies. (3) The sad part in de middle is elicited by the moment Ellie “gets told” in the hospital that she cannot have a baby: the posterior probabilities for a sad response rise briefly above chance level. After a short while, the couple picks up where they left off, and the distinction between the probabilities of a happy and sad response increase again. Over time, Carl and Ellie grow old and although they are still very happy with their lives together (4; Carl and Ellie dance together-scene), Carl realizes after having looked at an old photo (5) that much time has passed and their lives have not turned out the way they had hoped for. Eventually, we see Ellie in a hospital bed and at (6) she has just given back to Carl the book in which they had saved all their planned adventures. The posterior probabilities of a sad response rise above chance level from time to time and the happy response does not clearly dominate anymore.

The first and main part of the *Up* video contains predominantly happy content, and at a certain point in the video, the content becomes clearly less happy, while the level of sad content increases. This allowed us to demonstrate content validity of our method, by applying a classifier that was trained on the four emotion categories to the EEG data obtained during viewing the *Up* video, as well as to illustrate the application of the method with high temporal resolution. Fig 5 shows the posterior probabilities that the emotional response was happy or sad for every second of the *Up* video, averaged across participants and 500 repetitions (for illustrative purposes, we do not show the fear and disgust time courses in Fig 5; for the probabilities of the response being classified as each of the four emotions see Appendix K in S1 Supplementary Material. Classification of the emotional response is based on the same multi-class model in both figures, hence the only difference between the figures is the visibility of the fear and disgust time courses).

Fig 5 shows that although the emotional response of participants was mainly happy throughout the video as reflected by the estimated posterior probabilities, this response clearly decreased in the middle and also towards the end (while sadness is showing the opposite pattern), tracking the main ups and downs in the narrative.

## Discussion

Although many studies revealed that emotions and their dynamics have a profound impact on cognition and behavior, it has proven difficult to unobtrusively measure these emotions at a high temporal resolution. In the current study, our objective was to distinguish between emotional experiences using EEG in order to be able to continuously track dynamic changes in the emotional response over time. We investigated how accurately we could classify the experiences labelled as happy, sad, fear, and disgust which were naturally elicited by viewing various videos, based on the distinct patterns of frequency distributions observed in the EEG data. In addition, we illustrated how this method of classifying emotions can be applied on a moment-by-moment basis in order to track the emotional response elicited by viewing a movie clip dynamically over time.

The results showed that the classifiers were able to generalize the distinctions between emotions to new data well above chance level. We obtained a mean accuracy of 58% (vs. 25% chance level) when differentiating between all four emotions, and between 71 and 82% (vs. 50% chance level) when differentiating pairwise between specific emotions. Fear and disgust were shown to be the most difficult to distinguish based on the mean attained accuracy of just under 72%. This is in agreement with the self-report ratings of the videos showing that the videos which were meant to elicit fear, also elicited disgust to some extent; more so than videos which were meant to elicit the other emotions. Nevertheless, presenting our set of videos appeared to be a natural and reliable way to elicit specific emotional experiences consistently among participants. This is demonstrated by the ratings being specifically increased for the emotion that the videos targeted to elicit, and the high agreement between ratings reflecting participants’ similar feelings during viewing the multimodal dynamic stimuli.

Hence, we have demonstrated that we can distinguish between the specific emotional experiences happy, sad, fear, and disgust, and validated that the specific emotions that the videos were meant to elicit, corresponded with what the participants described to have experienced during viewing the videos. Thus, even though we cannot answer fundamental questions about the existence of (basic or specific) emotions in the brain [34], the results do suggest that representations of these emotional experiences, described as happy, sad, fear and disgust by our participants, can be distinguished in EEG data.

One potential issue is that, even though we targeted specific emotional experiences, the more general underlying dimensions of valence and arousal may (partly) also underlie our results. Since the four emotions in the current design do not sample the affective space of valence (from negative to positive) and arousal (from calm to excited) evenly, it is not possible to examine whether valence and arousal processes (partly) drive our results. Nevertheless, the data suggests that valence and arousal cannot completely account for our results either: the emotions fear and disgust should be assigned to a very similar position, in the same quadrant (i.e., middle/ high arousal and negative valence) within the valence-arousal space, yet the classification model is well able to distinguish fear from disgust.

With the current results, we were able to inspect how the emotional responses actually differed from each other in terms of patterns of frequency distributions and topography, upon which the classification is based. Importantly, the current approach allowed us to speculate about the interpretation of the differences in neural activity between emotional experiences, in terms of more general underlying processes that are known to be associated with these activation patterns (e.g., [35]). Alpha band activity (8–12 Hz), for example, has traditionally been related to the inverse of cortical activity. In a study combining EEG and fMRI registration in awake subjects at rest, alpha power correlated negatively with brain activity in parietal and lateral frontal cortices that are known to support attentional processes [36]. Indeed, many studies have shown that alpha activity is negatively associated with attention and task demands in general. More specifically, several EEG studies have shown that a decrease in posterior alpha power was associated with an increase in emotional arousal (e.g., [37] [38], but see [39] [40]).

Our results seem to be in line with these observations: we found that activity in the alpha frequency band was increased for the sad response, but reduced for the fear response predominantly at centro-posterior sites, in comparison to the other emotional responses. These activity patterns potentially reflect that attention and arousal are more strongly engaged for fear, but that they are attenuated for sad responses.

While alpha band activity mainly distinguished between the sad and fear response, activity in the higher frequency ranges distinguished happy and disgust from other emotions. Inspecting the scalp topography of the distinctions in these higher frequencies for happy and disgust, showed that the differences were rather local (instead of widespread) and peripheral. This suggests that these distinctions between emotions may in fact reflect muscle activity. Although we did not record electromyogram (EMG) from the relevant facial muscles, activity from the temporal and frontal muscles represents the most common form of EMG activity that is picked up by EEG (mainly in the higher frequency bands). Contraction of these muscles is produced by jaw clenching and raising eyebrows respectively, which the EEG picks up near the active muscles at the periphery of the scalp [41]. We could therefore speculate that increased high frequency activity at temporal sites elicited by disgust videos could have been caused by clenching the jaws, whereas reduced activity at temporal and frontal sites for the happy response may reflect reduced tension in the jaws and less frowning (as sad videos also appeared to elicit more frontal high frequency activity, potentially related to more frowning).

A side effect of having used multimodal stimuli to elicit emotions naturally could have been that participants displayed facial expressions corresponding with the elicited emotions (they were not asked to actively supress them). This, however, should not pose a problem, and may even work to our advantage if this kind of muscle activity from the facial expressions naturally occurs with the elicited emotions, and thus can be used in combination with brain activity to distinguish between emotional responses. That is, for purposes of decoding, it does not matter very much whether the signals that are used originate from the brain, the face, or from elsewhere within the body.

These findings demonstrate the value of the approach of retaining the frequencies that are present in the data as features to base classification of emotional experiences on. Particularly, when classification of emotions would be investigated in future studies with other (audio-visual) stimuli, differences in classification accuracies may occur because of the specific stimuli that are used. In other words, the use of different stimuli across studies very likely will result in different classification accuracies across studies, that may not be generalizable. However, with the current approach we could still assess the overlap between studies in terms of emotion-specific patterns of frequency distributions in the EEG data and their topography, reflecting interacting component psychological processes, despite any differences in stimuli and classification accuracies. That is, the emotion-specific EEG patterns on which the classification is based, may nevertheless be generalizable. Beyond offering insight into the processes underlying the differences between specific emotional experiences, this will ultimately aid generalization of the results and enable application of monitoring emotions over time in practice.

There have been some earlier attempts to classify specific emotions using EEG, but these studies have taken a different approach. Murugappan, Nagarajan, and Yaacob [42] aimed at a maximal classification of the emotions happy, surprise, fear, disgust, and neutral based on statistical features that were extracted from the EEG signal. Their entropy measure performed well at emotion classification, but leaves the distinctions between emotions uninterpretable. Using different stimuli, Lin and colleagues [43] presented their participants music to elicit emotions differing in valence and arousal. They averaged the frequencies present in the EEG data into five frequency bands (delta, theta, alpha, beta, gamma) and found that, based on asymmetries in oscillations, frontal and parietal electrode pairs were the most relevant in attaining the maximum classification accuracy. However, these authors did not attempt to classify specific emotions per se but rather the putative underlying dimensions of valence and arousal. Moreover, neither of these previous studies has shown whether it is possible to track emotions dynamically over time with their classification approach.

A unique feature of our study is the inclusion of an illustration of how this method of classifying emotions can actually be applied to relatively unobtrusively monitor emotional responses on a moment-by-moment basis. This tracking of the emotional experience is important, because emotions are, in essence, momentary experiences [23]. In the current study, participants viewed a movie clip from the animated movie *Up* that was especially included because of its complete story arc, in order to track dynamic changes in the emotional response elicited by viewing the movie clip, over time. We used a classifier that was trained on videos with happy, sad, fear and disgust content to estimate the average happy and sad response across participants, second-by-second during the movie clip. It appeared that the emotional response, which was estimated based on the EEG data, was able to accurately track the main ups and downs of the narrative, demonstrating content validity. In other words, this illustrates that our classification approach can be generalized to other videos that are not limited to eliciting mainly one emotion to an extreme extent, at a high temporal resolution. Further research is however needed in order to confirm the differentiation of emotional responses over time for a more diverse set of dynamic stimuli.

With the methodology advanced here, future research could address how the evolving emotional experience over time, as measured using EEG, relates to subsequent cognition and behavior without interfering with or disrupting the emotional experience under investigation. The implications of being able to implicitly measure people’s emotional response are numerous, and valuable in many contexts in which one is concerned with how a given stimulus is experienced over time. The user experience can provide information about attractiveness and appreciation in a variety of contexts ranging from clinical settings to consumer settings such as the consumption of digital media (such as movies, TV shows, broadcasted sports events), gaming, and online shopping.

To summarize, in the current study we elicited the emotional experiences happy, sad, fear, and disgust, and demonstrated that we could classify these using a multivariate approach. We retained all the frequencies that are present in the data, which allowed us to interpret the differences between emotions in terms of component psychological processes such as attention and arousal that are known to be associated with these activation patterns. The advantage of this approach is that it enables assessing the overlap between similar studies in terms of emotion specific patterns of frequency distributions in the EEG data and their topography. Additionally, we illustrated how this method of classifying emotional experiences can be applied on a moment-by-moment basis in order to relatively unobtrusively monitor dynamic changes in the emotional response as elicited by viewing a movie clip, over time.

## Supporting information

S1 Supplementary Material Details methods and additional analyses. (PDF)

## References

[1] Dolan RJ. Emotion, cognition, and behavior. Science. 2002;298:1191–1194. 10.1126/science.1076358 12424363

[2] Armony JL, Dolan RJ. Modulation of spatial attention by fear-conditioned stimuli: an event-related fMRI study. Neuropsychologia. 2002;40:817–826. 11900732 10.1016/s0028-3932(01)00178-6

[3] Ohman A, Flykt A, Esteves F. Emotion drives attention: Detecting the snake in the grass. J Exp Psychol. 2001;130:466–478. 10.1037/0096-3445.130.3.466 11561921

[4] Phelps EA. Human emotion and memory: Interactions of the amygdala and hippocampal complex. Curr Opin Neurobiol. 2004;14:198–202. 10.1016/j.conb.2004.03.015 15082325

[5] Elster J. Emotions and economic theory. J Econ Lit. 1998;36:47–74.

[6] Loewenstein G. Emotions in economic theory and economic behavior. Am Econ Rev. 2000;90:426–432.

[7] Peters E, Vastfjall D, Garling T, Slovic P. Affect and decision making: A “hot” topic. J Behav Decis Mak. 2006;19:79–85.

[8] Chen M, Bargh JA. Consequences of automatic evaluation: Immediate behavioral predispositions to approach or avoid the stimulus. Pers Soc Psychol Bull. 1999;25:215–224.

[9] Slovic P, Finucane ML, Peters E, MacGregor DG. The affect heuristic. Eur J Oper Res. 2007;177:1333–1352.

[10] Van ‘t Wout M, Chang LJ, Sanfey AG. The influence of emotion regulation on social interactive decision-making. Emotion. 2010;10:815–821. 10.1037/a0020069 21171756 PMC3057682

[11] Fisher RJ. Social desirability bias and the validity of indirect questioning. J Consum Res. 1993;20:303–315.

[12] Nisbett RE, Wilson TD. Telling more than we can know: Verbal reports on mental processes. Psychol Rev. 1977;84:231–259.

[13] Zajonc RB. Feeling and thinking: Preferences need no inferences. Am Psychol. 1980;35:151–175.

[14] Winkielman P, Berridge KC. Unconscious emotion. Curr Dir Psychol Sci. 2004;13:120–123.

[15] Dholakia UM, Morwitz VG. The scope and persistence of mere-measurement effects: Evidence from a field study of customer satisfaction measurement. J Consum Res. 2002;29:159–167.

[16] Feldman JM, Lynch JG. Self-generated validity and other effects of measurement on belief, attitude, intention, and behavior. J Appl Psychol. 1988;73:421–435.

[17] Plassmann H, Venkatraman V, Huettel S, Yoon C. Consumer neuroscience: Applications, challenges, and possible solutions. J Mark Res. 2015;52:427–435.

[18] Wager TD, Smith EE. Neuroimaging studies of working memory: A meta-analysis. Cogn, Affect, & Behav Neurosci. 2003;3:255–274. 10.3758/cabn.3.4.255 15040547

[19] Bartra O, McGuire JT, Kable JW. The valuation system: a coordinate-based meta-analysis of BOLD fMRI experiments examining neural correlates of subjective value. NeuroImage. 2013;76:412–427. 10.1016/j.neuroimage.2013.02.063 23507394 PMC3756836

[20] Lindquist KA, Wager TD, Kober H, Bliss-Moreau E, Barrett LF. The brain basis of emotion: A meta-analytic review. Behav Brain Sci. 2012;35:121–202. 10.1017/S0140525X11000446 22617651 PMC4329228

[21] Kassam KS, Markey AR, Cherkassky VL, Loewenstein G, Just MA. Identifying emotions on the basis of neural activation. PLoS One. 2013;8:e66032 10.1371/journal.pone.0066032 23840392 PMC3686858

[22] Kragel PA, LaBar KS. Decoding the nature of emotion in the brain. Trends Cogn Sci. 2016;20:444–455. 10.1016/j.tics.2016.03.011 27133227 PMC4875847

[23] Fredrickson BL, Branigan C. Positive emotions broaden the scope of attention and thought-action repertoires. Cogn Emot. 2005;19:313–332. 10.1080/02699930441000238 21852891 PMC3156609

[24] Gross JJ, Levenson RW. Emotion elicitation using films. Cogn Emot. 1995;9:87–108.

[25] Kahneman D, Fredrickson BL, Schreiber CA, Redelmeier DA. When more pain is preferred to less: Adding a better end. Psychol Sci. 1993;4:401–405.

[26] Do AM, Rupert AV, Wolford G. Evaluations of pleasurable experiences: The peak-and-end rule. Psychon Bull Rev. 2008;15:96–98. 18605486 10.3758/pbr.15.1.96

[27] Rugg MD, Coles MGH. Electrophysiology of mind: Event-related brain potentials and cognition Oxford, England: Oxford University Press;1995.

[28] Basar E, Basar-Eroglu C, Karakas S, Schurmann M. Oscillatory brain theory: A new trend in neuroscience. IEEE Eng Med Biol Mag. 1999;18:56–66. 10.1109/51.765190 10337564

[29] Knyazev GG. Motivation, emotion, and their inhibitory control mirrored in brain oscillations. Neurosci Biobehav Rev. 2007;31:377–395. 10.1016/j.neubiorev.2006.10.004 17145079

[30] Perria L, Rosadini G, Rossi GF. Determination of side of cerebral dominance with amobarbital. Arch Neurol. 1961;4:173–181. 13734520 10.1001/archneur.1961.00450080055006

[31] Davidson RJ, Fox NA. Asymmetrical brain activity discriminates between positive and negative affective stimuli in human infants. Science. 1982;218:1235–1237. 7146906 10.1126/science.7146906

[32] Harmon-Jones E, Gable PA, Peterson CK. The role of asymmetric frontal cortical activity in emotion-related phenomena: A review and update. Biol Psychol. 2010;84:451–462. 10.1016/j.biopsycho.2009.08.010 19733618

[33] Baumgartner T, Esslen M, Jäncke L. From emotion perception to emotion experience: Emotions evoked by pictures and classical music. Int J Psychophysiol. 2006;60:34–43. 10.1016/j.ijpsycho.2005.04.007 15993964

[34] Shackman AJ, Wager TD. The emotional brain: Fundamental questions and strategies for future research. Neurosci Lett. (In press). 10.1016/j.neulet.2018.10.012 PMC6370519 30473315

[35] Barrett LF, Wager TD. The structure of emotion: Evidence from neuroimaging studies. Curr Dir Psychol Sci. 2006;15:79–83.

[36] Laufs H, Krakow K, Sterzer P, Eger E, Beyerle A, Salek-Haddadi A, et al Electroencephalographic signatures of attentional and cognitive default modes in spontaneous brain activity fluctuations at rest. Proc Natl Acad Sci U S A. 2003;100:11053–11058. 10.1073/pnas.1831638100 12958209 PMC196925

[37] DeCesarei A, Codispoti M. Affective modulation of the LPP and α-ERD during picture viewing. Psychophysiology. 2011;48:1397–1404. 10.1111/j.1469-8986.2011.01204.x 21486291

[38] Simons RF, Detenber BH, Cuthbert BN, Schwartz DD, Reiss JE. Attention to television: Alpha power and its relationship to image motion and emotional content. Media Psychol. 2003;5:283–301.

[39] Aftanas LI, Varlamov AA, Pavlov SV, Makhnev VP, Reva NV. Time-dependent cortical asymmetries induced by emotional arousal: EEG analysis of event-related synchronization and desynchronization in individually defined frequency bands. Int J Psychophysiol. 2002;44:67–82. 11852158 10.1016/s0167-8760(01)00194-5

[40] Uusberg A, Uibo H, Kreegipuu K, Allik J. EEG alpha and cortical inhibition in affective attention. Int J Psychophysiol. 2013;89:26–36. 10.1016/j.ijpsycho.2013.04.020 23643563

[41] Goncharova II, McFarland DJ, Vaughan TM, Wolpaw JR. EMG contamination of EEG: spectral and topographical characteristics. Clin Neurophysiol. 2003;114:1580–1593. 12948787 10.1016/s1388-2457(03)00093-2

[42] Murugappan M, Nagarajan R, Yaacob S. Combining spatial filtering and wavelet transform for classifying human emotions using EEG Signals. J Med Biol Eng. 2011;31:45–51.

[43] Lin YP, Wang CH, Jung TP, Wu TL, Jeng SK, Duann JR, et al EEG-based emotion recognition in music listening. IEEE Trans Biomed Eng. 2010;57:1798–1806. 10.1109/TBME.2010.2048568 20442037

