# Supplementary material for: Implicit measurement of emotional experience and its dynamics
Source: PLoS One. 2019 Feb 5;14(2):e0211496. doi: 10.1371/journal.pone.0211496 (PMC6363176; doi:10.1371/journal.pone.0211496)
Supplement: S1 Supplementary Material — (PDF) [file pone.0211496.s001.pdf]

Appendix A. Details video clips

Supplementary Table 1. Selected video clips

Category	Video content from	Duration in s.	
Happy			
	500 Days of Summer	112	
	About Time	88	
	Love Actually	52	
	The Holiday	109	
	*Up**	200	
	Category mean	112.2	(54.6)
Sad			
	The Green Mile	135	
	The Help	106	
	Marley & Me	132	
	The Champ	110	
	The NeverEnding Story	107	
	Category mean	118.0	(14.3)
Fear			
	Anaconda	43	
	Annabelle	55	
	Friday the 13th – Part 2	96	
	Maze Runner – The Scorch Trials	123	
	The Ring	93	
	Category mean	82.0	(32.6)
Disgust			
	BuzzFeed Food	75	
	Fear Factor	80	
	Mr. Creostote	50	
	Pitch Perfect	22	
	Trainspotting	63	
	Category mean	58.0	(23.2)
Neutral (documentaries)			
	Wild Namibia	112	
	Modern Masters - Andy Warhol	100	
	The archers of Butan	105	
	China's high-speed train	106	
	Megastructures - Burj Khalifa Dubai	130	
	Category mean	110.6	(11.7)

Note: *The complete video duration of *Up* used for the illustration of tracking the emotional response over time was 261 seconds

Appendix B. Confirmation of elicited emotions by videos

In order to show that the videos we selected indeed predominantly elicited the experience of happy, sad, fear, and disgust (and not predominantly other emotions we did not inquire about), we ran an MTurk study with a free response format. 262 MTurkers each viewed four videos, with each video belonging to one of the four emotion categories (in total 20 videos with number of views per video varying from 50 to 56). Per video, we asked the participants which emotion they predominantly experienced during viewing the video. Before analyzing the results, two independent raters classified the MTurk responses as being valid responses or not, since some responses were clearly not valid (e.g., 'big snake', 'black guy speaking', 'teeth'). The proportion of agreement on validity of responses was .59, as expressed by Cohen's Kappa (good interrater agreement). The raters *agreed* that 54 out of the 273 unique responses should be considered *invalid*. Only the responses agreed on as invalid were deleted. Examples of disagreement across the two raters on validity of responses were 'crying', 'comradery', and 'ugly'. One participant was removed because he/she did not comment on any video, two participants were removed because they replied with 'bored' in response to all videos, and 23 because they replied with only one valid response across the four videos they watched (thus questioning the motivation of the responder). Single invalid responses were removed as well. The final analysis consisted of 933 responses (mean N across videos = 47, SD = 2.9, minimum N = 39 (*The Green Mile*), maximum N = 51 (*Trainspotting*)).

In order to analyze how many respondents answered with a label that corresponded to the hypothesized emotion the video predominantly elicited, we searched for the presence of the following words or word stems in the responses (see Supplementary Table 2 and 3).

Supplementary Table 2. Responses with synonyms for emotion category labels

Emotion category label	Corresponding words and word stems
Happy	happ*, joy*
Sad	sad*, grief, tearful
Fear	fear, afraid, fright*, scar*
Disgust	disgust

Supplementary Table 3. Responses with broader synonyms for emotion category labels

Emotion category label	Corresponding words and word stems
Happy	happ*, joy*, amuse*, excit*, cheer*, content, glad, glee, good, pleasant
Sad	sad*, depress*, upset, downcast, grief, tearful
Fear	fear, afraid, anxi*, creepy, fright*, scar*, spooked, terr*
Disgust	disgust, gross*, nausea, sick

In Supplementary Table 4, we present the percentage of respondents that answered with (synonyms for) the emotion category labels happy, sad, fear, and disgust, and in Table 5 we included the more broader synonyms for the emotion category labels. We additionally included 'love' as category label (word stems lov*, roman*), since there were a significant number of responses corresponding to this category (for the happy videos; *Love Actually* and *About Time*, 22% and 24% respectively, still considerably less than the happy responses, even when classified according to strict synonyms, 46% and 54% respectively). All other responses were categorized as 'other'. These other responses were quite diverse, ranging from labels belonging to the same 'emotion family' (most frequently), to labels describing a different feeling (more rarely). Examples of responses categorized as 'other' for the happy videos were: warm, hope*, touched, moved, inspir*, encouraged, sympathy, connected, confident, energetic. Examples of responses categorized as 'other' for the sad videos were: pity, sorrow, compassion, pain, remorse, empathy, sympathy, loss, impressed, cry* (cry* was not taken into account as sad response because it is a description of what occurs in some videos). Examples of responses categorized as 'other' for the fear videos were: thrill*, anticipation, nervous, unease, suspense, crying, curiosity, excitement, stress, panic, horr*, intrigued. Examples of responses categorized as 'other' for the disgust videos were: repulsed, unpleasant, annoy*, irritat*, nasty, ugly.

Supplementary Table 4. Percentage of responses for different emotion categories (strict synonyms)

Video category/ Response category	Happy	Sad	Fear	Disgust	Love	Other
Happy	55.98	4.27	0.43	0.43	11.11	27.78
Sad	0.43	73.39	2.58	0.00	0.86	22.75
Fear	0.00	2.12	69.07	1.69	0.00	27.12
Disgust	3.04	3.04	2.61	67.83	0.00	23.48

Supplementary Table 5. Percentage of responses for different emotion categories (including broader synonyms)

Video category/ Response category	Happy	Sad	Fear	Disgust	Love	Other
Happy	61.54	4.27	0.43	0.43	11.11	22.22
Sad	0.86	74.25	3.00	0.00	0.86	21.03
Fear	1.69	2.12	76.27	1.69	0.00	18.22
Disgust	5.65	3.04	2.61	72.17	0.00	16.52

The results in the tables show that even in a free response format, the participants accurately label the predominantly experienced emotions as happy, sad, fear and disgust (corresponding to the hypothesized elicited emotion). The tables show the average percentage of responses across videos within an emotion category, but this was also true for every single video. Thus, we show that asking the participants to indicate the extent to which they felt happy, sad, fear, and disgust on a five-point scale, produces highly similar results to an open response format question.

Appendix C. Detailed statistical analyses

Manipulation check. We first checked whether the videos were indeed effective in eliciting the targeted emotional response in the participants based on self-reported ratings of the emotions during viewing the videos (see Appendix H for results of the manipulation check and Supplementary Table 10). We started with verifying whether video category label had an effect on ratings of the different experienced emotions using a MANOVA. This main test was followed up by performing a repeated measures ANOVA for each of the categories of experienced emotions, to investigate whether the ratings of one specific experienced emotion were indeed higher for the videos that targeted the corresponding emotional response, compared to videos that targeted other emotional responses. Video category label served as IV (happy, sad, fear, disgust, neutral), and rating averaged across the five videos belonging to the category label served as DV. In addition to testing the effect of video label on the extent to which participants reported experiencing a particular emotion, we tested whether the four experienced emotions were rated differently within one video category. We checked whether the experienced emotion that was targeted by the videos, received a higher rating compared to the experienced emotions that were not targeted, by performing repeated measures ANOVAs for each of the video category labels (except for neutral), with experienced emotion as IV (happy, sad, fear, disgust), and rating averaged across the five videos belonging to the category label as DV. In order to check whether all participants experienced the emotions to a similar extent during viewing of the videos, we computed the intraclass correlation coefficient (ICC) for each of the experienced emotion categories that were rated using a two-way mixed-effects model where we were interested in the absolute agreement between raters.

Classification analyses. After transforming the EEG data obtained during viewing of the videos to the frequency domain, we standardized (i.e., z-transformed) the data for every participant, electrode, and frequency across all videos. Further analyses consisted of two parts with multiple stages.

In the first part of the analysis we investigated how accurately we could classify the emotional experience that was elicited by viewing a variety of videos, based on the patterns of frequency distributions in the EEG data. This part of the analysis consisted of three stages.

The first stage was feature selection. Using a subset of the observations (all the videos for all participants), we selected features (i.e., electrodes, frequencies) that were the most informative in distinguishing the emotions, in order to reduce dimensionality of the data and provide insight into the differentiation of emotional responses. We started with randomly

selecting two videos out of the five videos, for each of the emotions, for each of the participants. Using these two videos for each emotion, we created contrasts between emotions to find the most informative features. For each participant, and for each electrode and frequency, we determined the mean activity across the two videos for each emotion. From this mean, for each emotion, we subtracted the mean of the other three emotions, combined from six videos. This results in a value that indicates how distinctive that electrode and frequency for a participant is regarding one emotional response versus the average of the other three emotional responses. In order to detect similarities in these distinctions across participants, we computed for every emotion one-sample t-tests across participants, for every electrode and frequency. Finally, in order to discover the most distinctive and thus informative features, we selected for every emotion the electrodes and frequencies with the 10% highest t-values (see Appendix E for similar results with 5% and 20% features: robustness check 2). We used the most informative features from all emotions combined in the subsequent stages.

Second, in order to associate the emotion labels with patterns in the EEG data, we used classification models in the form of SVMs with a linear kernel function. Having detected the most informative features, we only used these electrodes and frequencies from the EEG data as features in the SVM models for the remainder of the observations that were not used for feature selection (i.e., three videos for each emotion and participant). After having divided the remaining observations into 10 different folds with approximately equal representations of the emotions, the training stage employed 9 out of the 10 folds, referring to 9/10 of these remaining observations. Since we were not only interested in how well the emotions can be classified in general, but also in a comparison of the specific emotions one-by-one, we computed six two-class classification models for the six combinations of four emotions (happy-sad, happy-fear, happy-disgust, sad-fear, sad-disgust, fear-disgust). In addition, a multi-class classification model was estimated containing all four emotion categories. We approached the multi-class classification problem using the Error-Correcting Output Codes (ECOC) framework (Dietterich and Bakiri, 1995[1]), in which multiclass learning is reduced to multiple (SVM) binary learners. We used an Error-Correcting Output Codes (ECOC) classifier as implemented by Matlab (R2016b; Statistics and Machine Learning Toolbox) with a one-versus-all coding design in the case of classifying all four emotions.

[1] Dietterich, T. G., & Bakiri, G. (1995). Solving multiclass learning problems via error-correcting output codes. *Journal of Artificial Intelligence Research, 2*, 263-286.

All this resulted in training seven classifiers to associate the emotion labels with patterns in the EEG data. We did not intend to classify a ‘neutral response’ from the neutral videos that were presented between emotion blocks, but including the neutral category in the classifiers yielded similar results (see Appendix F for robustness check 3).

Third, we tested the trained classifiers on the remaining data. We iterated stage two and three ten times for all possibilities of leaving out one of the ten folds. We then evaluated the ability of the classification models to generalize the distinction between emotions to new data by calculating the percentage of accurately predicted emotion labels across observations from all ten folds (i.e., the out of sample generalization accuracy).

An important quality of the analysis design is that the same data is not used twice, for reduction of the data and training/ testing of the models. This means however, that the specific subset of observations that we used in the different analysis stages, are of influence on the features that are selected as the most informative ones in the first stage, and also ultimately on the accuracy of the SVM models in the final stage. Since we did not want to select the most informative features based on specific videos, we decided to randomly select a subset of videos (two per emotion and per participant) for feature selection, leaving the remainder of the observations for the train and test stage. In the end, we repeated all of the stages described above 500 times, with for each repetition a different random selection of observations for the feature selection and thus the train and test stage (see Appendix G for robustness checks regarding the number of repetitions). These 500 repetitions resulted in a distribution that approximates all the possible values that our measures of interest (the generalization accuracies in the final stage) can adopt, and thus rules out a selection bias as explanation of our results.

In the second part of the analysis, we classified happy, sad, fear, and disgust emotional experiences from the happy, sad, fear, and disgust videos, and thereafter applied the trained classifier to EEG data obtained during viewing of the complete *Up* video on a moment-by-moment basis, in order to track the probability that the emotional response is happy or sad over the time course of the video. In this analysis, we first removed the happy video *Up* from the data, as well as the sad video *The Help* which had the lowest average rating for a sad response, the video *Anaconda* which had the lowest average rating for a fear response, and the video *Pitch Perfect* which had the lowest average rating for a disgust response (see Appendix H), in order to keep the number of videos equal across emotion conditions and to prevent training a classification model biased towards one emotional response. With the remaining four videos for happy, sad, fear, and disgust, we proceeded through stage one of feature selection and stage two of training a classifier similarly to in the

first part of the analysis (except that the contrast in feature selection was now based on one video per emotion per participant instead of on two). Instead of testing the classifier at the third stage, we used the classifier to compute the posterior probabilities that the emotional response was happy or sad for every second of the *Up* video, and we averaged these probabilities across participants. We repeated all of the stages 500 times also for this second part of the analysis, with for each repetition different observations used for the feature selection and training (and prediction) stage. This again resulted in a distribution that approximates all the possible values that our measure of interest (here, posterior probabilities) can adopt, and thus rules out a selection bias as explanation of the results. For the probabilities of the response being classified as each of the four emotions see Appendix K.

Appendix D. Robustness check 1: Similar duration of analyzed segments across emotions

In order to empirically test whether classification was potentially biased due to the difference in duration of the videos (thus, possibly a systematically different signal to noise ratio in the EEG data) across emotion conditions, we conducted a robustness check. We repeated the complete analysis with only the data in response to the final 22 seconds of each video (i.e., the duration of the shortest video) in order to eliminate the effect of duration difference across emotion conditions. We find that the resulting classifiers were still able to generalize the distinction between all four emotional experiences to new data well above chance level, even when merely the final 22 seconds of the data were taken into account for all videos. Hence, we can exclude that different durations of the videos across emotions biased the classification results. We can also conclude from these results that there is indeed information specific to the emotions in the extra length of the videos from the sad and happy categories, since especially this distinction is less accurately predicted by the final 22 seconds compared to the complete data (i.e., approximately a 10 percentage-point decrease).

Supplementary Table 6. Mean and percentiles for the distributions of out of sample generalization accuracies across 500 repetitions, using only the final 22 seconds of each video

Classifier		Mean	Min.	2.50%	25%	50%	75%	97.50%	Max.
Fear	Disgust	64.91	53.15	58.56	62.16	64.86	67.34	71.62	76.58
Sad	Disgust	76.03	66.22	70.27	73.87	76.13	78.15	81.98	84.68
Sad	Fear	70.60	61.26	64.41	68.47	70.72	72.52	76.58	81.08
Happy	Disgust	73.02	63.06	66.67	70.72	72.97	75.23	79.73	81.53
Happy	Fear	69.95	62.16	63.51	67.57	69.82	72.52	76.13	77.93
Happy	Sad	67.50	59.01	60.81	65.32	67.57	69.82	73.87	75.68
Multi-class (all 4 emotions)		46.10	38.51	40.99	43.92	46.17	47.97	51.58	54.28

Appendix E. Robustness check 2: Changing the number of features that are used for classification

In order to explore the influence of the number of features used in the classifiers, we varied the percentage of selected features between 5%, 10%, and 20% of the highest *t*-values. The results in the tables below show that this only had a marginal impact on the ultimate out-of-sample accuracies.

Supplementary Table 7. Mean and percentiles for the distributions of out of sample generalization accuracies across 500 repetitions, using the 5 percent most informative features

Classifier		Mean	Min.	2.50%	25%	50%	75%	97.50%	Max.
Fear	Disgust	70.30	57.21	63.06	68.02	70.27	72.52	77.48	80.18
Sad	Disgust	81.14	73.42	76.13	79.28	81.08	82.88	86.04	87.84
Sad	Fear	74.16	65.32	68.47	72.07	74.10	76.13	80.18	83.33
Happy	Disgust	76.44	67.57	71.17	74.32	76.58	78.38	81.98	85.59
Happy	Fear	75.41	67.57	70.27	73.42	75.23	77.48	81.08	83.33
Happy	Sad	78.49	69.37	72.97	76.58	78.38	80.63	83.78	85.59
Multi-class (all 4 emotions)		56.37	48.65	51.35	54.73	56.31	58.11	61.26	64.19

Supplementary Table 8. Mean and percentiles for the distributions of out of sample generalization accuracies across 500 repetitions, using the 20 percent most informative features

Classifier		Mean	Min.	2.50%	25%	50%	75%	97.50%	Max.
Fear	Disgust	71.82	61.71	65.77	69.82	72.07	73.87	77.03	79.73
Sad	Disgust	81.67	74.77	77.03	80.18	81.53	83.33	86.49	88.74
Sad	Fear	74.97	66.22	69.37	72.97	75.23	77.03	80.63	83.33
Happy	Disgust	77.32	69.37	71.62	75.23	77.48	79.73	82.88	85.59
Happy	Fear	76.92	68.92	71.17	74.77	76.58	78.83	81.98	84.23
Happy	Sad	77.90	67.12	72.07	76.13	77.93	79.73	82.88	87.84
Multi-class (all 4 emotions)		58.05	50.68	53.60	56.53	58.11	59.68	62.61	64.86

Appendix F. Robustness check 3: Classifying the neutral videos viewed in-between emotion blocks

Including ‘neutral’ as an emotion category yielded out of sample generalization accuracies for the classifiers that were in a similar range to the classifiers currently described in the main manuscript. This can be regarded as another robustness check since the neutral trials were not presented sequentially in one block to participants, but instead presented between the other emotion blocks, yet the classifiers were able to recognize these separate neutral trials as a category distinct from the trials belonging to the other emotion categories. Supplementary Table 9 shows that happy versus neutral was the most difficult to distinguish based on the classifier’s attained accuracy. This is in agreement with the ratings of the videos showing that the videos which were meant to be neutral, clearly elicited more of a happy feeling (mean rating across neutral videos 2.43), than a sad, fear, and disgust feeling (mean rating across neutral videos respectively 1.34, 1.20, 1.12) (see Appendix H Results manipulation check, Supplementary Table 10).

Supplementary Table 9. Mean and percentiles for the distributions of out of sample generalization accuracies across 500 repetitions, including neutral as category

Classifier		Mean	Min.	2.50%	25%	50%	75%	97.50%	Max.
Disgust	Neutral	77.69	68.02	72.52	75.68	77.93	79.28	83.33	86.49
Fear	Neutral	74.09	65.32	68.92	72.07	74.32	76.13	79.73	83.33
Fear	Disgust	72.08	61.26	65.77	69.82	72.07	74.32	78.38	80.63
Sad	Neutral	74.75	66.22	69.82	72.97	74.77	76.58	79.73	81.53
Sad	Disgust	81.98	74.32	77.03	80.18	81.98	83.78	86.49	88.74
Sad	Fear	75.32	65.77	69.82	72.97	75.23	77.48	81.08	83.33
Happy	Neutral	63.84	54.50	57.21	61.71	63.96	66.22	70.27	72.07
Happy	Disgust	77.53	70.27	72.52	75.68	77.48	79.28	82.43	84.23
Happy	Fear	77.13	70.72	72.07	75.23	77.03	78.83	82.43	85.14
Happy	Sad	78.53	70.72	72.97	77.03	78.83	80.18	83.33	87.84
Multi-class (all 5 emotions)		49.84	44.14	45.23	48.29	49.91	51.35	54.59	56.58

Appendix G. Repetition of the analysis with different observations in the different stages to rule out a selection bias

An important quality of the analysis design is that the same data is not used twice in the different analysis stages, but this also means that the specific subset of observations that we used in the stages are of influence on the outcome. In the first stage, a specific subset of observations influences the features that are selected as the most informative ones, and in the final stage it influences the out of sample generalization accuracy of the SVM models. Since we did not want to select the most informative features based on specific videos, we decided to randomly select a subset of videos for feature selection. For each of the 37 participants, we selected for each of the 4 emotions, randomly 2 videos out of the 5 videos ('ABCDE'), which leaves us with 10 possible selections of videos per emotion per participant (AB, AC, AD, AE, BC, BD, BE, CD, CE, DE) for feature selection. This means that there are 10^(4x37) possibilities available to split the data for feature selection versus training/ test set, rendering an extensive computation of accuracies for all possible video selections unfeasible. We therefore repeated the complete analysis including the three stages multiple times in order to investigate the influence of using a specific subset of observations in the first stage, leaving the remainder of the observations for the final stages. For every repetition of the analysis, we selected a different random subset of observations in the first stage for feature selection (keeping the numbers across participants and emotions constant), thus a different subset of observations remained for the second and third training and testing stage. We compared the distributions of accuracies for 10 repetitions, 50, 100, 500, 1000 and 5000 repetitions of the complete analysis (see Supplementary Figure 1). Although the distributions of generalization accuracies (from the final stage) did not change drastically from 10 to 5000 repetitions, at 500 repetitions, the distribution was virtually indistinguishable from 5000 repetitions. Therefore, we repeated the complete analysis with all of the stages 500 times, each time with a different selection of observations for the stages, to be able to create a distribution that approximates all the possible values that our measures of interest (i.e., t-values in the feature selection stage, generalization accuracy in the final testing stage) can adopt, and thus to rule out a selection bias.

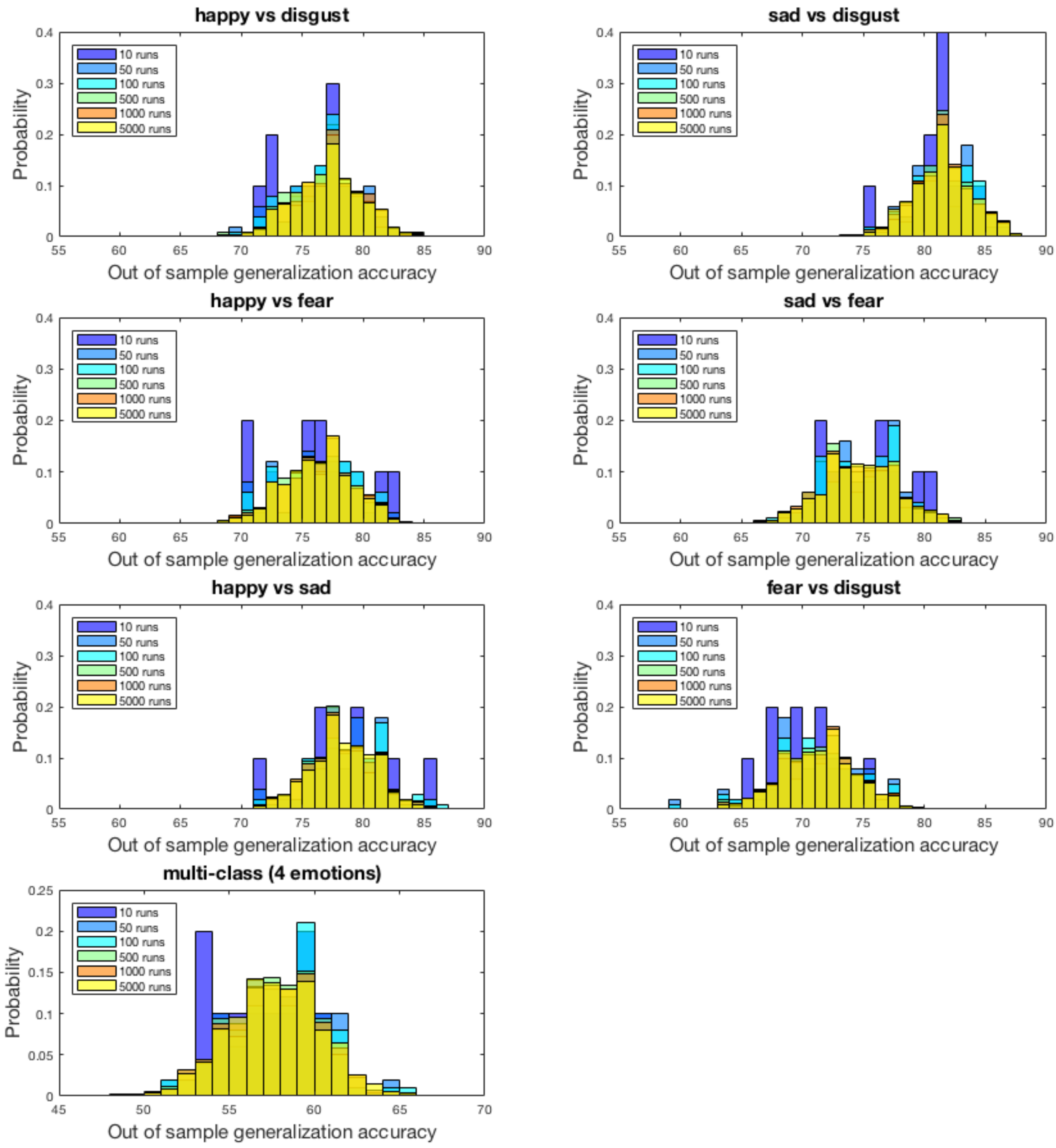

Supplementary Figure 1. For each of the classifiers, the distributions of generalization accuracies for 10, 50, 100, 500, 1000, and 5000 repetitions (runs) of the analyses with different subsets of observations in the analyses stages

Appendix H. Results manipulation check

Because we wanted to verify the videos' effectiveness in eliciting the specific emotional responses in our participants (i.e., manipulation check), we asked participants to complete a questionnaire about the previously viewed videos after we finished the EEG data collection. Participants had to indicate for each video the extent to which they felt happy, sad, fear, and disgust during the video on a scale from one (not felt at all e.g., happy) to five (felt extremely e.g., happy).

As expected, the MANOVA indicated an interaction between video label (i.e., the target emotion) and the emotion participants reported they actually experienced, using Pillai's trace ($V = 0.98$, $F(9, 28) = 129.89$, $p < .001$). The follow-up ANOVAs showed that the ratings of a specific experienced emotion were higher for the videos that targeted this particular emotional response, compared to videos that did not target this emotional response ($F_{\text{Happy ratings}}(4,144) = 158.72$, $F_{\text{Sad ratings}}(4,144) = 121.32$, $F_{\text{Fear ratings}}(4,144) = 78.90$, $F_{\text{Disgust ratings}}(4,144) = 186.71$, all $p < .001$, with follow-up Bonferroni corrected pairwise comparisons also $p <.001$ for the videos targeting the specific emotional response compared to the videos targeting the other emotional responses). That is, we checked that participants reported to feel happier during viewing happy videos compared to during viewing sad, fear, disgust, or neutral videos (and more sad during sad videos, compared to happy, fear disgust, and neutral videos, etc.). The F-values are presented in the Supplementary Table 10 together with ratings of the experienced emotions for the corresponding videos. The second set of ANOVAs revealed that the experienced emotion that was targeted, received higher ratings compared to the experienced emotions that were not targeted ($F_{\text{Happy videos}}(3,108) = 341.85$, $F_{\text{Sad videos}}(3,108) = 145.19$, $F_{\text{Fear videos}}(3,108) = 67.51$, $F_{\text{Disgust videos}}(3,108) = 153.58$, all $p <.001$, with follow-up Bonferroni corrected pairwise comparisons also $p <.001$ for the targeted experienced emotion compared to the three other non-targeted emotions). That is, we also checked that participants reported to feel happier during viewing happy videos than they reported to feel sad, fear and disgusted (and feel more sad during sad videos, than to feel happy, fear, and disgusted, etc.). The intraclass correlation coefficient (ICC) of each of the experienced emotion categories for the mean of multiple ratings was $ICC_{\text{Happy}} = .98$, $ICC_{\text{Sad}} = .98$, $ICC_{\text{Fear}} = .97$, $ICC_{\text{Disgust}} = .99$.

Based on the results of the ANOVAs and the ICCs, we conclude that the emotional response that the video targeted to elicit, is indeed the emotion that the participants predominantly experienced during viewing of the videos. These results suggest that the EEG activity averaged across the duration of the videos, is representative of a happy, sad, fear, and

disgust response, respectively, and that we can use this data to functionally localize specific emotion-related activity patterns.

Supplementary Table 10. Mean (SD) ratings of videos across the 37 participants

	Happy	Sad	Fear	Disgust	RM ANOVA $F(3,108)$
Happy videos					
500 Days of Summer	4.11 (0.81)	1.05 (0.33)	1.00 (0.00)	1.00 (0.00)	
About Time	3.73 (1.07)	1.46 (0.69)	1.16 (0.55)	1.11 (0.46)	
Love Actually	3.97 (0.99)	1.95 (1.05)	1.16 (0.60)	1.05 (0.33)	
The Holiday	3.76 (1.01)	1.38 (0.86)	1.19 (0.46)	1.05 (0.23)	
Up	4.11 (0.81)	2.22 (1.42)	1.22 (0.67)	1.00 (0.00)	
Category mean	3.94 (0.94)	1.61 (0.87)	1.15 (0.46)	1.04 (0.20)	341.85*
Sad videos					
The Green Mile	1.24 (0.55)	3.54 (1.12)	2.08 (1.23)	1.38 (0.86)	
Marley & Me	1.19 (0.46)	3.86 (1.23)	1.70 (1.02)	1.22 (0.63)	
The Champ	1.11 (0.31)	3.89 (1.02)	1.84 (1.09)	1.22 (0.67)	
The Help	1.24 (0.49)	2.95 (1.31)	1.41 (0.90)	1.22 (0.53)	
The NeverEnding Story	1.19 (0.46)	3.70 (1.22)	2.49 (1.35)	1.30 (0.57)	
Category mean	1.19 (0.46)	3.59 (1.18)	1.90 (1.12)	1.26 (0.65)	145.19*
Fear videos					
The Ring	1.22 (0.53)	1.38 (0.72)	3.68 (1.38)	2.57 (1.30)	
Friday the 13th	1.22 (0.58)	1.65 (1.18)	3.38 (1.34)	2.08 (1.23)	
Anaconda	1.30 (0.62)	1.86 (0.98)	2.62 (1.32)	2.05 (1.27)	
Anabelle	1.11 (0.39)	1.43 (0.93)	3.38 (1.40)	1.30 (0.66)	
Maze Runner	1.16 (0.50)	1.22 (0.63)	3.32 (1.36)	2.59 (1.40)	
Category mean	1.20 (0.53)	1.51 (0.89)	3.28 (1.36)	2.12 (1.17)	67.51*
Disgust videos					
BuzzFeed Food	1.81 (1.05)	1.24 (0.55)	1.49 (0.69)	3.46 (1.37)	
Fear Factor	1.62 (0.95)	1.54 (0.90)	2.00 (1.20)	4.54 (0.77)	
Mr. Creostote	1.86 (1.11)	1.19 (0.52)	1.14 (0.48)	3.57 (1.19)	
Pitch Perfect	2.19 (1.41)	1.11 (0.39)	1.08 (0.28)	3.16 (1.17)	
Trainspotting	1.43 (0.96)	1.24 (0.55)	1.43 (0.83)	4.43 (0.87)	
Category mean	1.78 (1.10)	1.26 (0.58)	1.43 (0.70)	3.83 (1.07)	153.58*
Neutral videos					
Wild Namibia	2.49 (0.99)	1.68 (1.11)	1.43 (0.77)	1.19 (0.57)	
Modern Masters	2.30 (1.15)	1.30 (0.66)	1.11 (0.39)	1.19 (0.52)	
The archers of Butan	2.62 (1.11)	1.43 (0.69)	1.11 (0.39)	1.11 (0.39)	
China's high-speed train	2.49 (1.19)	1.22 (0.58)	1.16 (0.44)	1.05 (0.33)	
Megastructures	2.24 (1.32)	1.05 (0.23)	1.19 (0.46)	1.05 (0.23)	
Category mean	2.43 (1.15)	1.34 (0.65)	1.20 (0.49)	1.12 (0.41)	/
RM ANOVA $F(4,144)$	158.72*	121.32*	78.90*	186.71*	

Note: * $p < .001$

Appendix I. Selection of the most distinctive features for classification

In the subsequent figures, we present how often the features were selected, and thus were used in training and testing the classifiers taking as criterion the 10% highest *t*-values per emotion, expressed as a percentage across the 500 repetitions.

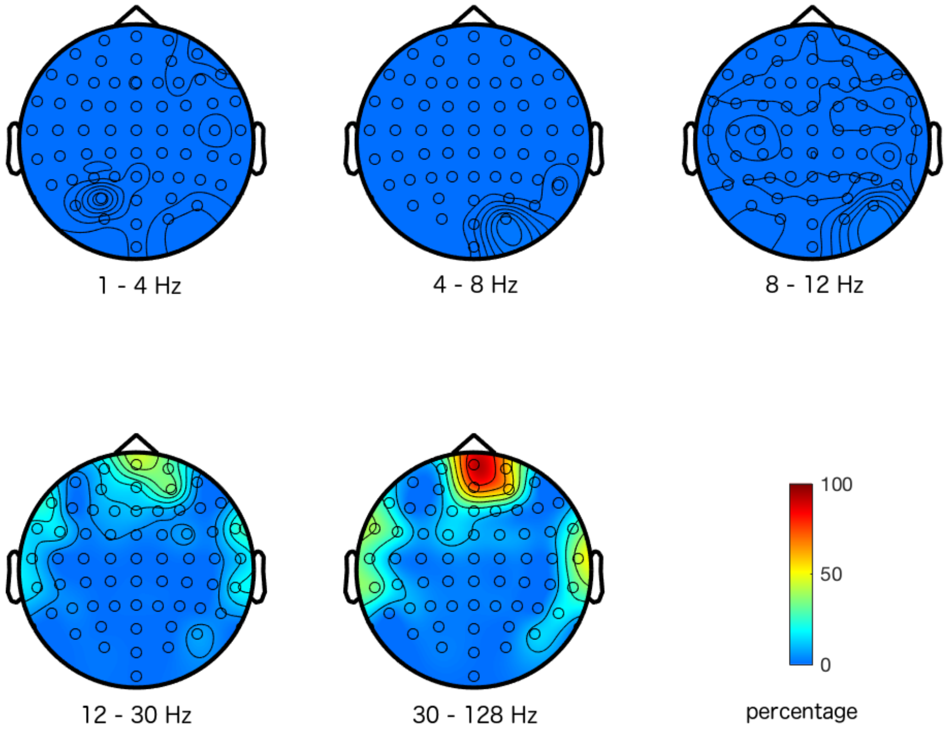

Supplementary Figure 2. Color maps of how often the features are selected as most distinctive for the happy response and thus used in training and testing the classifiers. The colors represent the percentage of repetitions that the specific frequencies (mentioned below the different scalp maps), and electrodes (across the heads) were selected as the features with 10% highest *t*-values for the happy contrast.

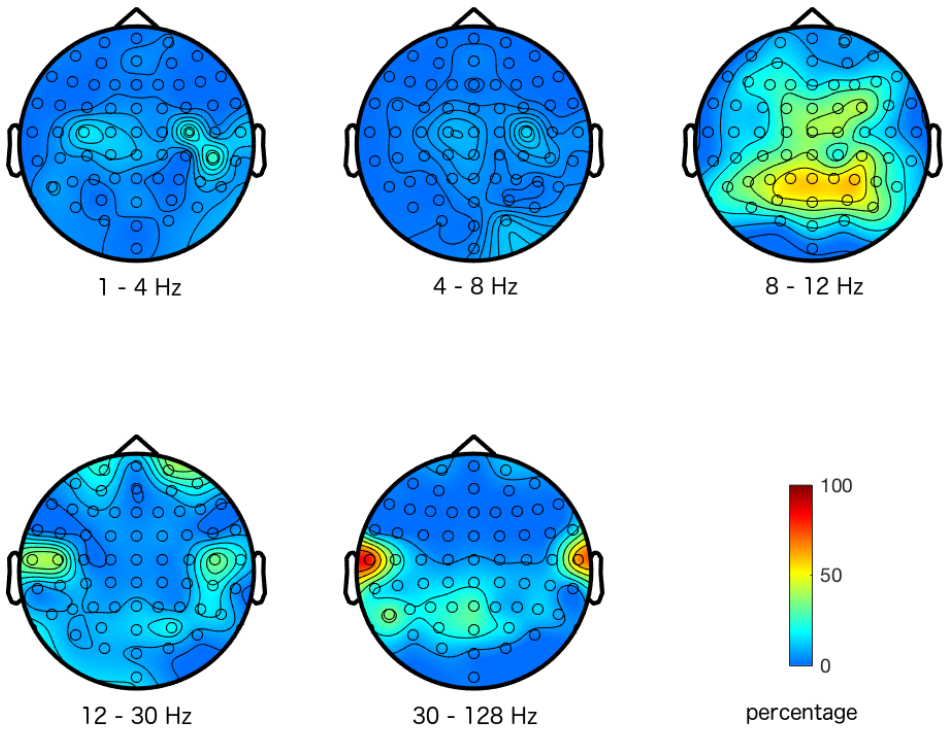

Supplementary Figure 3. Color maps of how often the features are selected as most distinctive for the sad response and thus used in training and testing the classifiers. The colors represent the percentage of repetitions that the specific frequencies (mentioned below the different scalp maps), and electrodes (across the heads) were selected as the features with 10% highest *t*-values for the sad contrast.

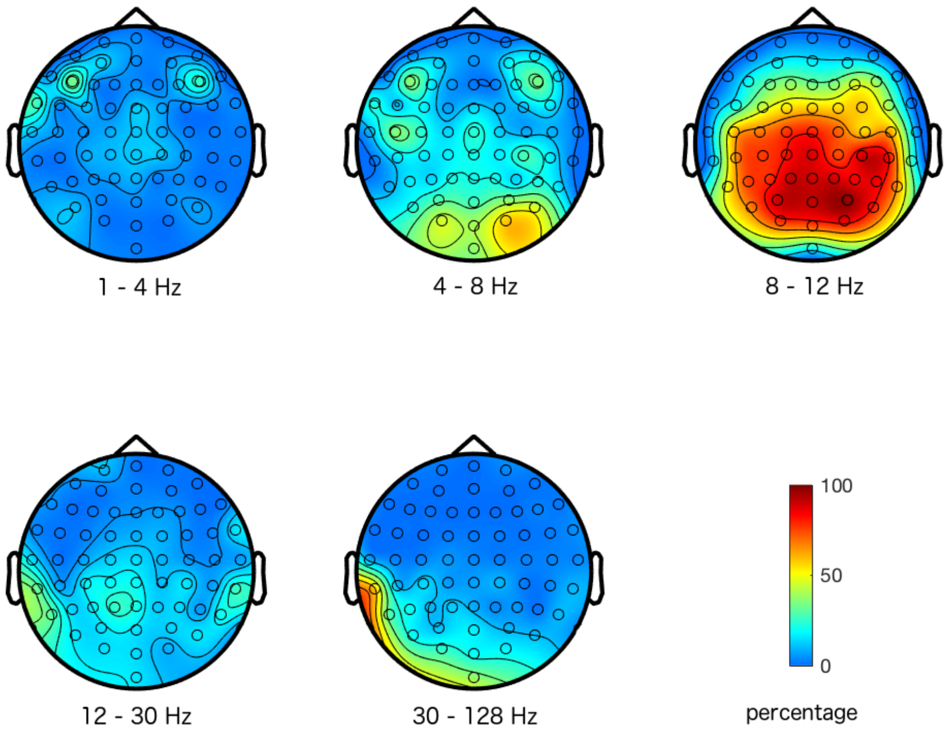

Supplementary Figure 4. Color maps of how often the features are selected as most distinctive for the fear response and thus used in training and testing the classifiers. The colors represent the percentage of repetitions that the specific frequencies (mentioned below the different scalp maps), and electrodes (across the heads) were selected as the features with 10% highest *t*-values for the fear contrast.

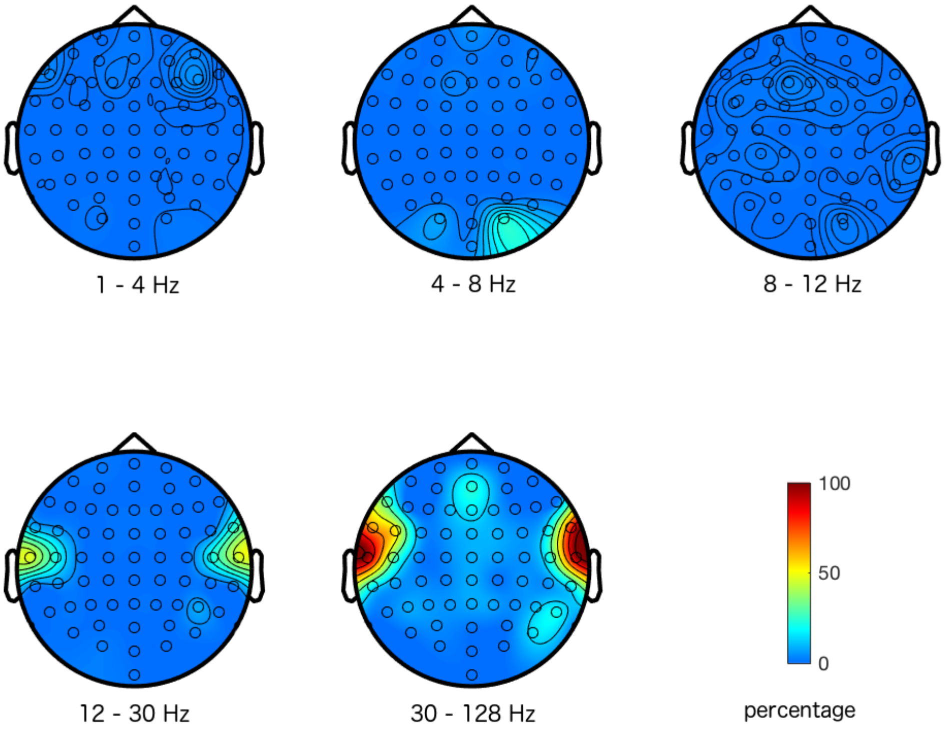

Supplementary Figure 5. Color maps of how often the features are selected as most distinctive for the disgust response and thus used in training and testing the classifiers. The colors represent the percentage of repetitions that the specific frequencies (mentioned below the different scalp maps), and electrodes (across the heads) were selected as the features with 10% highest *t*-values for the disgust contrast.

Appendix J. Permutation-based benchmark for models' generalization accuracies

In order to determine whether there is a significant amount of information present in the EEG data that is emotion-specific, we created a permutation-based benchmark to compare the performance of the models against, instead of simply testing the models' performance to the theoretical (a priori) chance level. We repeated the training stage and the testing stage, but with random permutation of the emotion labels for the training set, and with testing of the models' performance on the true labels, keeping the folds similar to the original classification procedure. We repeated this procedure ten times with different random permutations of the training data, and averaged the generalization accuracy across these ten repetitions. We repeated this analysis also 500 times in a similar fashion to our original analysis (i.e., each repetition we used different cases for the feature selection and training/ testing stages), resulting in similar distributions of generalization accuracies as for the original data. Computing the out of sample generalization accuracy for the 500 repetitions using a model trained on random data showed that the median of the distribution was around theoretical chance level, and the maximum achieved accuracy (i.e., 54.46%) was still clearly lower than the minimum of the models trained on original data (not taking into account the multi-class model, for which the randomly trained model's accuracy achieved maximally 26.98%) (see Supplementary Table 11). This means that the two distributions with generalization accuracies from the original models and with generalization accuracies from the random models did not overlap. Non-parametric and distribution free Kolmogorov-Smirnov tests confirmed this (all D=1.00, p <.00001), hence the EEG data contained a significant amount of information that is class-specific and could be used in order to predict the emotional responses from participants based on their EEG activity.

Supplementary Table 11. Mean and percentiles for the distributions of out of sample generalization accuracies across 500 repetitions, using permuted emotion labels

Classifier		Mean	Min.	2.50%	25%	50%	75%	97.50%	Max.
Fear	Disgust	49.93	47.16	47.88	49.19	49.91	50.70	51.98	52.48
Sad	Disgust	49.96	46.67	47.79	49.21	49.95	50.72	52.12	53.06
Sad	Fear	49.90	47.16	47.57	49.17	49.82	50.68	52.12	53.78
Happy	Disgust	49.92	47.21	47.88	49.19	49.86	50.63	52.07	53.29
Happy	Fear	50.02	46.80	47.84	49.35	50.05	50.72	52.07	54.46
Happy	Sad	49.94	46.85	47.66	49.14	49.98	50.77	51.98	53.24
Multi-class (all 4 emotions)		25.01	23.40	23.76	24.55	25.02	25.41	26.33	26.98

Appendix K. Emotional response classification for *Up* as happy, sad, fear or disgust

Supplementary Figure 6 shows the posterior probabilities that the emotional response was classified as happy, sad, fear or disgust for every second of the *Up* video, averaged across participants and 500 repetitions (for illustrative purposes, we did not show the fear and disgust time courses in Fig 5 in the main paper). Classification of the emotional response is based on the same multi-class model in both figures, hence the only difference between the figures is the visibility of the probability that the response is classified as fear and disgust.

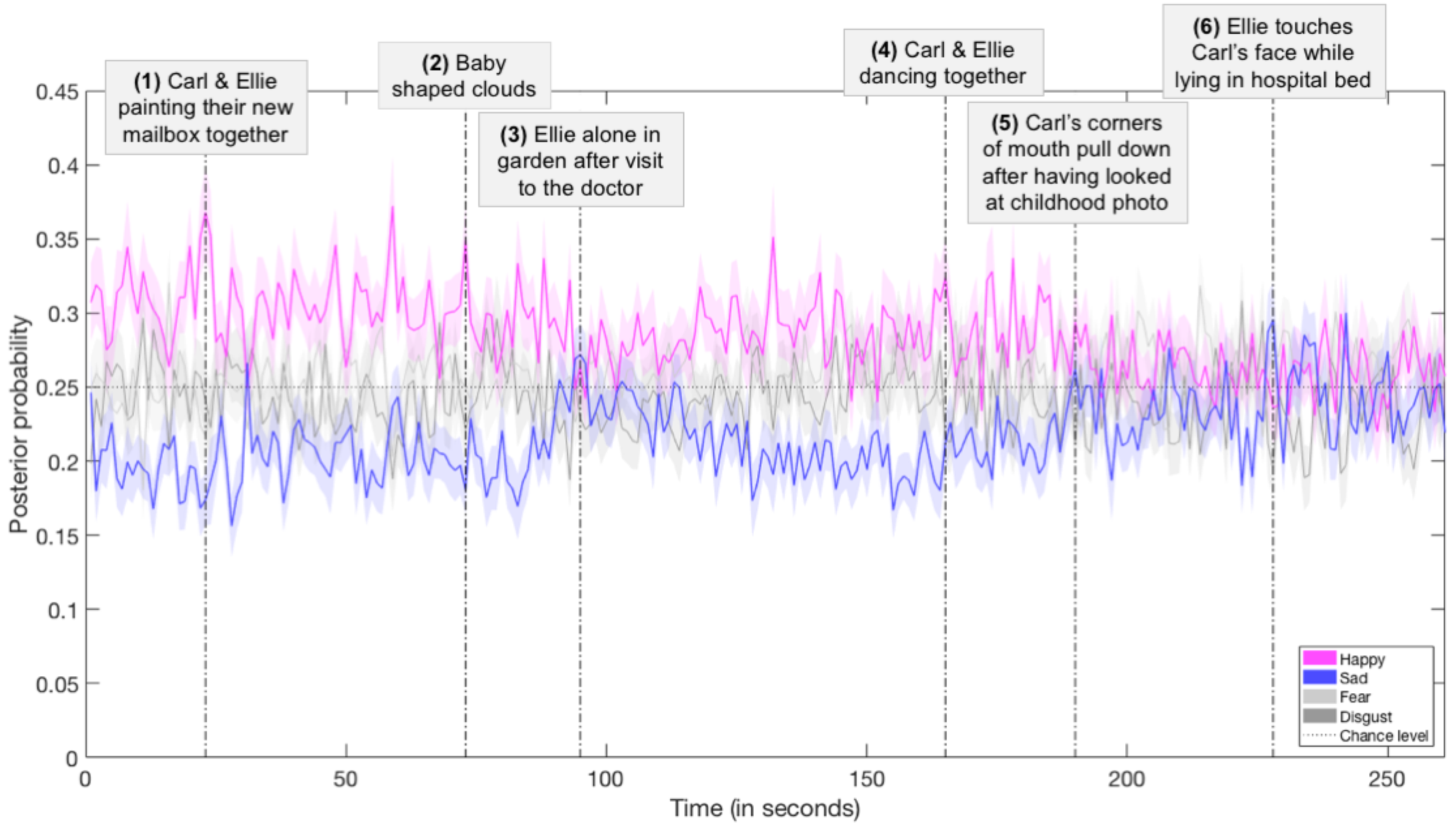

Supplementary Figure 6. Dynamics of posterior probabilities for *Up* with four emotions. Averaged across participants and 500 repetitions, with different observations used in the feature selection and classifier training stages across repetitions. The shaded areas indicate the standard deviation across repetitions.
